# Discrete turn strategies emerge in information-limited navigation

**Published:** 2026-06-22

**Authors:** Jose M. Betancourt, Matthew P. Leighton, Thierry Emonet, Benjamin B. Machta, Michael C. Abbott

**Affiliations:** 1 Department of Physics and Quantitative Biology Institute, Yale University, New Haven, CT, USA; 2 Molecular, Cellular, and Developmental Biology, Yale University, New Haven, CT, USA

## Abstract

Navigation up a smooth sensory gradient is one of the simplest behavioural tasks, and some organisms solve it by making continuous adjustments to their course. Bacteria instead employ a variety of discrete strategies, including run and tumble motion, direction reversals, and turns by specific angles. Here we ask what drives the choice of these strategies, framing the problem as maximising up-gradient speed with a given amount of sensory information per unit time. We find that, without directional information on which way to turn, behavioural strategies that take discrete actions perform better than gradual steering. As the amount of information is increased, we see a series of transitions between optimal strategies, including a shift from direction reversals to fully re-orienting tumbles. Among more complex re-orientation strategies, we show that discrete turn angles are best, and observe transitions in the number of angles employed by the optimal strategy. More broadly, such emergent simplicity in behaviour is a tractable example of a widespread phenomenon in which biology chooses a discrete solution, despite the underlying physics being continuous.

## Introduction

Many micro-organisms navigate by switching between a small set of discrete behavioural states in response to continuous changes in their sensory input. Run and tumble is one such strategy, alternating between swimming as straight as possible, and suddenly picking a new direction at random [1]. Other organisms swim backwards and forwards [2], or make right-angle turns [3]. Why do they do these things, instead of making continuous changes? And how do they choose which strategy to adopt? In this paper we offer an explanation based only on the constraint that they have limited sensory information.

These questions about navigation belong to a wide class of related questions about why biology chooses discrete solutions where the underlying physics is continuous, or nearly so. For example, the human tongue can make a continuum of vowel sounds, but every language discretises this space to give meaning to a small number of phonemes. Why do this, and what controls the number? In the salamander, thousands of retinal ganglion cells have the thresholds at which they turn on or off set to just three values [4]. Why not use intermediate settings? More microscopically, most ion channels are either open or closed. Why not allow a graded current through the membrane? None of these questions are at the atomic scale, where the ultimate discreteness of the underlying physics would enter.

What all of these systems have in common is that they involve information processing. Mathematically, optimising an objective function containing a measure of information often leads to a discrete solution [5–10]. If evolution has performed optimisation, then perhaps the simple discrete solutions seen in its products are in a sense deliberate. Perhaps they aren’t compromises based on the difficulty of making control systems out of proteins, nor the result of a trade-off between performance and complexity, but rather, signs that something close to the ideal solution has been attained. Getting access to these these ideas about simplicity, and what we term emergent discreteness, is our main motivation for studying toy models of a behavioural task.

The task we study is navigation up a fixed sensory gradient. This task is solved by many organisms, and the case of *E. coli* in a shallow gradient of attractant has been shown to be close to optimal at using the information it does receive to execute its chosen strategy [11]. In this bacterium, sensory proteins bind to attractant molecules, and these binding events lead to changes in its internal chemical state, which in turn regulates the motors that drive the flagella [12]. The noise in every step of this process limits how much information flows from heading to behaviour. We focus on this end-to-end information rate, to obtain a framework in which to study how limited information shapes behavioural strategies, while remaining agnostic to organism-specific details.

However, while bacteria can typically only measure how the concentration changes along their path, larger organisms can directly measure spatial gradients across their bodies. For example, fruit flies compare signals between their two antennae, allowing them to measure the instantaneous gradient perpendicular to their line of travel. It will be important to make a distinction between such directional information and the non-directional kind collected by *E. coli*. While both may be expressed in bits per second, knowing which way to steer is qualitatively different, and changes what strategies are preferred.

What we find is that if the sensor does not provide directional information, then continuous steering is always worse than sudden changes of direction. As the amount of sensory information varies, for instance because the gradient steepness changes, we see various transitions of which strategy is best. Further, if we allow arbitrary actions, then the optimal strategy employs a discrete set of turn angles. These results are an example of how discreteness emerges from optimisation with limited information, a phenomenon which may explain many otherwise surprising discrete actions in biology.

## Results

For simplicity we start with navigation in two dimensions, with an agent swimming at some fixed speed $v_{0}$, at an angle θ away from the desired direction. Its mean speed towards the goal is then $v=v_{0}\langle cos\theta \rangle_{\theta }$. The angle θ suffers rotational diffusion $D_{r}$, which tends to randomise the heading in time of order $1/D_{r}$. To make progress, this passive noise from diffusion must be counteracted by a control system.

We assume the the agent senses some information about its instantaneous heading, and uses this to produce a controlled action $d\theta_{c}$. The active and passive contributions are added to obtain the full update dθ for time dt. Our system can be sketched as follows:


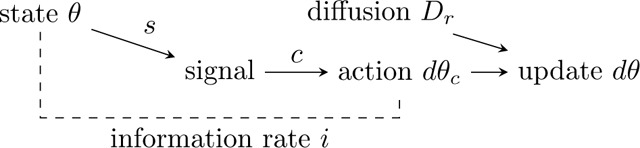



We measure the fidelity of the control response by the mutual information rate from state to action, $i=I\left(\Theta ;d\Theta_{c}\right)/dt$. In any real system both sensing and control are noisy, and both place limits on the response. We do not model them separately, and describe a strategy by some function of heading θ with Poisson or diffusive noise. However, to study strategies available without directional sensing, we will impose that the response be the same for θ and −θ. This can be viewed as inserting s(θ)=|θ| while optimising over c. (We also treat s(θ)=sign(θ) in appendix C.5.)

To find the control strategy which maximises mean climbing speed at a given information rate, we introduce a trade-off parameter γ>0, to arrive at the following optimisation problem:

(1) $$\underset{c}{max}\left(v/v_{0}-\gamma \;i\right).$$

**Figure 1:**
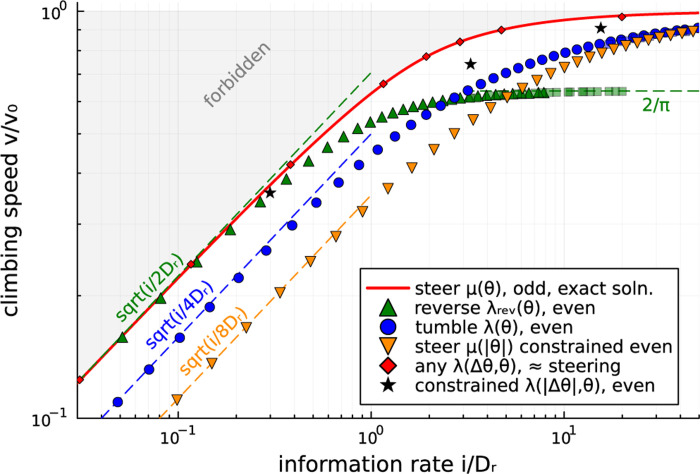
Performance of strategies for two-dimensional navigation, on the speed-information plane. The continuous steering strategy achieves the highest performance, provided the sign of heading θ is visible (red). Among strategies not using such directional information, i.e. those described by even functions of θ, fully re-orienting instantaneous tumbles (blue) perform well at high information rates, but at lower rates reversing (green) needs half as much information for the same speed. All low-information limits scale as $v∼\sqrt{i}$, and high-information limits are $v/v_{0}\to 1$ except reverse, which is limited to $v/v_{0}<2/\pi \approx 0.64$. Information is measured in nats, thus the axis is in nats per rotational diffusion time. Plot points are numerical, and square points use ansatz $\lambda_{\text{strong}}(\theta )$. Black stars are the three solutions shown in figure 3.

Finding the optimal control strategy c for many different values of γ gives us a frontier on the speed-information plane (figure 1). We do this first for some particular classes of controller, before turning to the general case of an arbitrary instantaneous response.

### Steering vs. tumbling, in two dimensions

We begin by comparing two behaviours: *steering*, which continuously updates the heading at rate μ, and *tumbling*, which picks a new heading at random with rate λ. We divide the evolution of the heading dθ in time dt into a controlled part $d\theta_{c}$ and Gaussian noise dW from diffusion:

(2) $$d\theta =d\theta_{c}+\sqrt{2D_{r}}dW\;\;\;d\theta_{c}=\mu (\theta )dt+\Delta \theta dJ(\lambda (\theta ))+\sqrt{2D_{c}}dW^{'}.$$

Here dJ is a Poisson jump process, Δθ∼U(−π,π) is the change in heading after a tumble, and $D_{c}$ is controller noise. The equivalent Fokker-Planck equation is

$$\frac{dp(\theta )}{dt}=-\lambda (\theta )p(\theta )+\int \frac{d\theta }{2\pi }p(\theta )\lambda (\theta )\;(\text{tumbling})\;\;-\frac{d[\mu (\theta )p(\theta )]}{d\theta }+\left(D_{c}+D_{r}\right)\frac{d^{2}p(\theta )}{d\theta^{2}}\;(\text{steering}\;\&\;\text{noise}).$$

Given the description of the strategy by λ(θ), μ(θ) and $D_{c}$, we can solve dp(θ)/dt=0 to obtain the steady-state distribution. This distribution p(θ) can be viewed as a population average, or the long-time average of one individual. It allows us to compute the mean climbing speed, $v/v_{0}=\langle cos\theta \rangle_{\theta }=\int d\theta \;p(\theta )\;cos\theta$.

The information rate we use is mutual information between the heading θ and the controlled update $d\theta_{c}$, per time dt. For tumbling alone, this is

(3) $$i=I\left(\Theta ;d\Theta_{c}\right)/dt={\left\langle \lambda (\theta )log\frac{\lambda (\theta )}{{\left\langle \lambda \left(\theta^{'}\right)\right\rangle }_{\theta^{'}}}\right\rangle }_{\theta }.$$

This form can be derived by considering the two possible actions, p(tumble∣θ)=λ(θ)dt and p(run∣θ)=1−λ(θ)dt. The formula for continuous steering can be derived by allowing small steps left or right of angle ±α at rates $\beta_{\pm }(\theta )=D_{c}/\alpha^{2}\pm \mu (\theta )/2\alpha$. We give more detail in appendix C.1 (and another approach in E.2), but the result is an information rate proportional to the variance of μ(θ):

(4) $$i={\left\langle {\left[\mu (\theta )-{\left\langle \mu \left(\theta^{'}\right)\right\rangle }_{\theta^{'}}\right]}^{2}\right\rangle }_{\theta }/4D_{c}.$$

The optimised solutions to (1) always have controller noise $D_{c}=D_{r}$, with the magnitude of steering μ(θ) varying to produce high and low information rates. Explicit controller noise is not needed for the tumble strategy, as the intrinsic noise of the Poisson process plays the same role.

**Figure 2:**
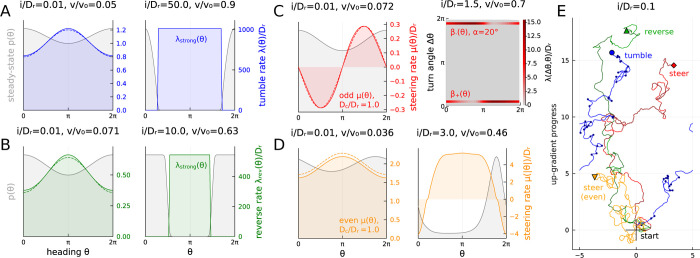
Numerical optimal strategies for run & tumble, steering, and reversing. (A) Tumble rate λ(θ) in blue, and steady-state p(θ) in grey, for a low-information case, and a high-information case using $\lambda_{\text{strong}}(\theta )$. (B) The run-reverse strategy achieves $\sqrt{2}$ times the speed at similar information rate $i/D_{r}\approx 0.01$, by acting at half the rate of the tumble strategy. But its high-information case saturates at speed 2/π, when p(θ) is uniform on −π/2≤θ≤π/2. (C) Steering rate μ(θ) in red, and corresponding p(θ) in grey. Second panel translates turn rate μ(θ) and controller noise $D_{c}=D_{r}$ to Poisson rate λ(Δθ,θ) using two small angles Δθ=±α. (D) Steering rate μ(|θ|), assuming the sign of θ is not observable. At low information rate, $i/D_{r}\approx 0.01$, the agent always turns in one direction but modulates its turning speed. At high information rate, this strategy creates stable & unstable fixed points at some $\pm \theta_{⋆}$. (A-D) Dashed lines are analytic results at low information, from the text. (E) Sample trajectories for four strategies, all with $i/D_{r}\approx 0.1$ (times 0<t<50 in units $D_{r}=v_{0}=1$, wrapped to −5<x<5). More trajectories are shown in appendix A.

Using equations (2)–(4) in objective (1), we now have all the pieces to find optimal tumbling and steering strategies. We show some of them in figure 2, and discuss their properties here before turning to more general strategies in later sections.

For tumbling, our setup is very similar to that used by Mattingly et. al. [11]. They drew a frontier on the speed-information plane, at low information rates, and were able to experimentally place *E. coli* at about 70% of optimal performance, given the information they actually receive. In a shallow gradient, these bacteria receive around 1% of a bit per run. In this regime the tumble rate is only slightly modulated, as shown in figure 2A:

$$\lambda (\theta )=D_{r}\left[1-\sqrt{4i/D_{r}}cos\theta +𝒪\left(i/D_{r}\right)\right].$$

The resulting performance is $v/v_{0}\approx \sqrt{i/4D_{r}}$ for $i\ll D_{r}$, which is a dashed line in figure 1. Here we assume that the turns are instantaneous, although real *E. coli* spend perhaps 10% of their time tumbling [13]. In appendix B.2 we derive the effect of time penalty τ per tumble on the solution, and show that a large penalty $\tau >0.69/D_{r}$ is needed for the rank order of strategies to change.

In the opposite limit, when information is plentiful, our problem approaches the one studied by Strong et. al. [14]. They asked what strategy gives the fastest climbing, with a finite time penalty τ per tumble, but no cost of information. They found a deterministic strategy: the agent should always tumble when $|\theta |>\theta_{\text{thresh}}$. To achieve high speed when tumbles are quick, $\tau \ll 1/D_{r}$, the threshold is low $\left|\theta_{\text{thresh}}\right|\ll 1$, describing a behaviour in which the agent tumbles many times in succession until landing on a nearly-uphill heading. Similar behaviour is seen in the solution to our problem with τ=0 but a large finite information rate, $i\gg D_{r}$. We find that the following strategy, shown in figure 2A, is a reasonable approximation:

(5) $$\lambda_{\text{strong}}(\theta )=\left\{\begin{array}{ll} 0 & |\theta |<\theta_{\text{thresh}}\\ \lambda_{max} & \text{else}. \end{array}\right.$$

Numerically, we can trace out the entire range between the low- and high-information limits, giving the blue points on figure 1.

Turning now to study continuous changes of heading, we optimise both the steering rate μ(θ) and the controller noise $D_{c}$. The solution always has $D_{c}=D_{r}$, and it’s possible to solve for μ(θ) exactly in terms of Mathieu special functions. We give details in appendix C.2, but the leading term at low information rate is:

$$\mu (\theta )=-\sqrt{8iD_{r}}sin\theta +𝒪\left(i/D_{r}\right).$$

This limit $i\ll D_{r}$ has performance $v/v_{0}\approx \sqrt{i/2D_{r}}$, and we plot the full curve on figure 1. In the high-information limit the heading is tightly constrained, |θ|≪1, producing a simple linear control problem.

We see that the steering strategy has uniformly higher performance than tumbling. But an important difference is that it is exploiting the sign of θ, in a way that tumbling could not. The optimal tumble rate is an even function, λ(θ)=λ(−θ), but the optimal steering rate is odd, μ(θ)=−μ(−θ). A strategy in which the agent is always able to steer towards the correct direction may be relevant for a ship with a poor compass, or an organism large enough to sense the local gradient vector. But if the agent can only observe its rate of up-gradient motion — as for instance a ship measuring on the depth of water, or a bacterium sensing the rate of change of concentration — then it must be ignorant of the sign, and hence μ(θ) must be even.

We can impose this symmetry constraint μ(θ)=μ(−θ) when finding solutions. Without access to the sign of θ, the performance of steering is worse than tumbling, showing that discrete strategies can out-perform continuous adjustments. In figure 1, the low-information limit is $v/v_{0}\approx \sqrt{i/8D_{r}}$ for $i\ll D_{r}$. The strategy in this limit is to always turn in one direction, but slightly bias the rate:

$$\mu (|\theta |)=\pm 2D_{r}\left[1-\sqrt{2i/D_{r}}cos\theta +𝒪\left(i/D_{r}\right)\right].$$

At high information rates, the strategy is more interesting, see figure 2D. The optimal μ(|θ|) changes sign to create a pair of fixed points at $\pm \theta_{⋆}$, and the agent spends most of its time at whichever one is stable. For each such strategy, turning the other way gives equivalent performance, but moves across the gradient at speed $v_{0}\langle sin\theta \rangle_{\theta }$ with the opposite sign.

### Transitions between turn strategies

In the absence of directional information, we have seen that tumbles, i.e. turns by a randomly chosen angle Δθ∼U(−π,π), perform better than continuous steering. It is possible to do better by controlling the turn angle, and the best strategy at low information rates is in fact to *reverse* direction, Δθ=π. The Fokker-Planck equation for this strategy is

$$\frac{dp(\theta )}{dt}=-\lambda_{rev}(\theta )p(\theta )+\lambda_{rev}(\theta +\pi )p(\theta +\pi )+D_{r}\frac{d^{2}p(\theta )}{d\theta^{2}}$$

and the information rate is still (3). The optimal rate of reversal at low information rates is (appendix B.3, figure 2B)

$$\lambda_{rev}(\theta )=D_{r}/2\left[1-\sqrt{8i/D_{r}}cos\theta +𝒪\left(i/D_{r}\right)\right].$$

This strategy uses half as much information for the same speed as tumbling: $v/v_{0}\approx \sqrt{i/2D_{r}}$ for $i\ll D_{r}$. Curiously, in this limit reverse achieves the same performance as the steering solution μ(θ). But at high information rates, the best that it can do is to place all probability density within |θ|<π/2 (shown in figure 2B), leading to $\langle cos\theta \rangle_{\theta }=2/\pi \approx 0.64$. Hence figure 1 shows a crossover: a transition between reverse and tumble being the best strategy, among the three not using the sign of θ.

In appendix B we consider a *flick* strategy which uses right-angle turns, Δθ=±π/2. This strategy beats both tumble and reverse in a medium-information regime (around $i/D_{r}\approx 10$), but cannot exceed $\langle cos\theta \rangle_{\theta }=\sqrt{8}/\pi \approx 0.9$. Thus we see two transitions between simple discrete strategies as the amount of information available increases.

### Discrete angles from arbitrary turns

Instead of a fixed turn angle Δθ or a fixed distribution, we now look at the general case where λ(Δθ,θ) is the rate of initiating turns by Δθ from heading θ. All of the strategies already considered can be written as such a rate:

$$\lambda (\Delta \theta ,\theta )=\left\{\begin{array}{ll} \lambda (\theta )/2\pi & \text{tumble}\\ \delta (\Delta \theta -\pi )\lambda_{\text{rev}}(\theta ) & \text{reverse}\\ \delta (\Delta \theta -\alpha )\beta_{+}(\theta )+\delta (\Delta \theta +\alpha )\beta_{-}(\theta ) & \text{steering}. \end{array}\right.$$

Here we again approximate steering as turns by small angles Δθ=±α with rates $\beta_{\pm }(\theta )=D_{c}/\alpha^{2}\pm \mu (\theta )/2\alpha$, as shown in figure 2C. For the general case the Fokker-Planck equation reads

$$\frac{dp(\theta )}{dt}=\int d\phi [\lambda (\theta -\phi ,\phi )p(\phi )-\lambda (\phi ,\theta )p(\theta )]+D_{r}\frac{d^{2}p(\theta )}{d\theta^{2}}$$

and the information rate is

(6) $$i=\iint d\Delta \theta \;d\theta \;\lambda (\Delta \theta ,\theta )p(\theta )log\frac{\lambda (\Delta \theta ,\theta )}{\overset{‾}{\lambda }(\Delta \theta )}$$

where we define $\overset{‾}{\lambda }(\Delta \theta )=\int d\theta^{'}\;\lambda \left(\Delta \theta ,\theta^{'}\right)p\left(\theta^{'}\right)$.

Solving numerically for the optimal λ(Δθ,θ), we recover solutions equivalent to steering, which are shown as red points on figure 1. The close agreement on performance is numerical evidence that steering via μ(θ) is truly optimal; we hope to present analytic results on this question soon [15]. These unconstrained solutions obey λ(Δθ,θ)=λ(−Δθ,−θ), which aligns with an odd steering rate, μ(−θ)=−μ(θ); see appendix C.1 for examples.

**Figure 3:**
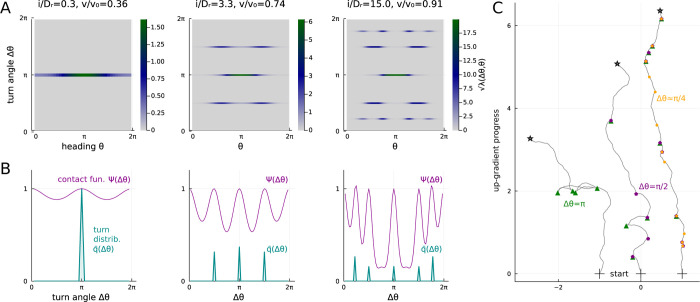
Discrete optimal strategies for three information rates. (A) We impose that rate λ(Δθ,θ) is even in Δθ, which implies that it is even in θ, i.e. the strategy ignores the sign of θ. At low information rate (left) we recover the reverse strategy, Δθ=π, but with increasing information it bifurcates to use three angles (centre), and then five (right). The two new strategies shown are faster than both reverse and tumbling: see the black stars on figure 1. (B) Below each rate, we plot the contact function Ψ(Δθ) and a mean distribution $\overset{‾}{q}(\Delta \theta )\propto \overset{‾}{\lambda }(\Delta \theta )$. The rate λ(Δθ,θ) is nonzero precisely where Ψ(Δθ)=1. Notice that Ψ(0)=1, but we do not allow turns of Δθ=0. (C) Sample trajectories for the same three strategies, all for times 0≤t≤7 (units $D_{r}=v_{0}=1$). Notice that in the second case, a flick turn by Δθ=±π/2 (purple) is often followed by a reversal, Δθ=π (green), when the flick happened to pick the wrong direction. This strategy isn’t quite run-reverse-flick [3], as one flick can follow another flick, each turn is independently chosen.

However, if we look for strategies not using the sign of θ, then something more interesting happens, shown in figure 3. At low information rates, we recover the reverse strategy, which is the true optimum. But as more information becomes available, the solution bifurcates to use two additional angles, Δθ≈±π/2. With more information, it bifurcates again to use five angles, and so on, but the distribution of turn angles remains discrete.

To see why this discreteness of Δθ emerges, we now formulate an augmented problem in which the Fokker-Planck equation dp(θ)/dt=0 is imposed as a constraint, along with the symmetry λ(Δθ,θ)=λ(−Δθ,θ), and the normalisation of p(θ). These are enforced by Lagrange multipliers, and thus we maximise the following 𝓛 with respect to p, λ, χ, φ, ξ:

$$𝓛=\langle cos\theta \rangle_{\theta }-\gamma i+\int d\theta \chi (\theta )\frac{dp(\theta )}{dt}+\varphi \left[1-\int d\theta \;p(\theta )\right]\;\;\;+\iint d\Delta \theta \;d\theta \;\xi (\Delta \theta ,\theta )[\lambda (\Delta \theta ,\theta )-\lambda (-\Delta \theta ,\theta )].$$

In addition to the three equality constraints, there are still two inequality constraints: p(θ)≥0 and λ(Δθ,θ)≥0. This last constraint plays a crucial role, as the equation of motion ∂𝓛/∂λ(Δθ,θ)=0 need only be satisfied where the constraint is slack, λ(Δθ,θ)>0. After some algebra, this equation of motion reads

$$\lambda (\Delta \theta ,\theta )/\overset{‾}{\lambda }(\Delta \theta )=e^{[\chi (\theta +\Delta \theta )+\chi (\theta -\Delta \theta )-2\chi (\theta )]/2\gamma }.$$

Taking the expectation value with p(θ) on both sides leads to Ψ(Δθ)=1, where we define

(7) $$\Psi (\Delta \theta )=\int d\theta \;p(\theta )e^{[\chi (\theta +\Delta \theta )+\chi (\theta -\Delta \theta )-2\chi (\theta )]/2\gamma }.$$

This contact function is analytic, and clearly has Ψ(0)=1. Thus it must either be constant, or else have Ψ(Δθ)=1 at a set of isolated points. In appendix D.4 we show that $\Psi^{''}(0)=-i/D_{r}<0$, ruling out the constant case. Hence λ(Δθ,θ) is zero except at a discrete set of angles Δθ. Figure 3 plots the contact function alongside the numerical solutions λ(Δθ,θ) used to find it, for three information rates.

A similar construction without the symmetry constraint leads to the contact function being $\Psi (\Delta \theta )=\int d\theta p(\theta )e^{[\chi (\theta +\Delta \theta )-\chi (\theta )]/\gamma }$. In the appendix we show that, when evaluated on steering solutions, this is 1 everywhere. The symmetry constraint may be thought of as changing a circular parameter space 0≤Δθ<2π into a line 0≤Δθ≤π with reflecting boundary conditions. Such fold lines, and other explicit boundaries of parameter space, played a crucial role in the emergence of discreteness in our work on Bayesian priors [8].

Related arguments for discreteness from analyticity have been made in channel capacity problems, see for instance [5, 8, 16], and there are some results about the number of points in the support of the solution [9, 10]. The maximisation problem we study here is more complicated, and is most similar to that of [7].

### Navigation in three dimensions

While some organisms do navigate on a two-dimensional plane, many swim freely in three dimensions. Here we show that the qualitative results above survive, and some are strengthened.

**Figure 4:**
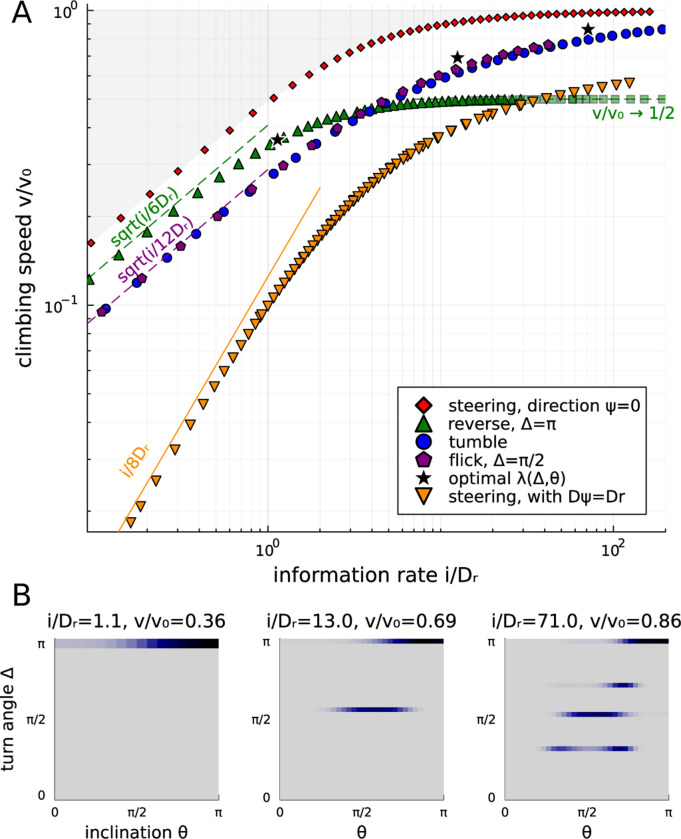
Navigation strategies in three dimensions. (A) Performance of tumble and reverse are qualitatively similar to figure 1, with different prefactors in the $v∼\sqrt{i}$ scaling at low information rates. Flick produces similar performance to tumble. The red steering points are a strategy which controls only the inclination θ, turning always towards the pole at θ=0. The orange points are a steering strategy in which the agent suffers diffusion $D_{\psi }$ in its roll angle ψ (in addition to diffusion of its heading, $D_{r}$), and this is much worse than not knowing the sign of θ in two dimensions, leading to performance v∼i at low information rates. (B) Optimal rates λ(Δ,θ) for a strategy allowed any turn angle Δ. The roll angle ψ is uncontrolled, hence Δ=π (flick) represents a turn to anywhere on the circle perpendicular to the current heading. As in figure 3 we see a progression from reverse to reverse & flick to something more complicated, but always using a discrete set of angles Δ.

The tumble and reverse strategies have obvious generalisations to three dimensions, and figure 4A shows their performance. At low information rates reverse is again faster, with $v/v_{0}\approx \sqrt{i/6D_{r}}$, and again becomes slower than tumbling at high information rates, now $v/v_{0}<1/2$.

Steering is more complicated, as the direction in which the agent turns is now a vector, tangent to the sphere of possible headings. The equivalent of making use of the sign of θ is now steering always towards the pole, θ=0. If this is possible, then the cost of controlling θ alone is low, producing high performance: the red points on figure 4A. However, the agent can also roll around its heading direction, which is described by an additional angle ψ. The equivalent of not knowing the sign of θ in two-dimensional steering is not knowing this roll angle, and this greatly degrades the value of steering. If we assume the roll angle suffers from diffusion with noise $D_{\psi }$, and take $D_{\psi }=D_{r}$ then what we see numerically is, very approximately,

$$v/v_{0}\approx i/8D_{r},\;i\ll D_{r}.$$

Analytically, we show in appendix E.4 that $v∼i^{s}$ with slope s≥1. This is a much steeper decline at low information rate than the discrete strategies, which all scale as $v∼\sqrt{i}$.

The flick strategy, making right-angle turns, performs much like tumble. One difference from two dimensions is that it now has limit $v/v_{0}\to 1$ at high information rates, because a succession of right-angle turns in three dimensions can bring the heading arbitrarily close to the pole. Here too we assume the roll angle is not controlled, hence each turn by Δ=π/2 places the heading new anywhere on a circle perpendicular to the original heading.

We can similarly study the general case where λ(Δ,θ) is the rate at which turns are initiated, placing the new heading somewhere on a circle angle Δ away from the old heading vector. Figure 4B shows what we see. At low information rates, all weight is on Δ=π, the reverse strategy. With more information, turns of Δ≈π/2 appear too, and there are further bifurcations to use additional angles. But the distribution of turn angles remains discrete, much like in two dimensions (figure 3).

## Conclusion

In this navigation problem we see two kinds of discreteness emerge. First, among the strategies not using directional information, sudden actions like tumble and reverse perform better than smooth steering (figures 1, 4A). Second, if arbitrary turn angles are allowed, the optimal strategy uses a discrete set of angles (figures 3, 4B). This has parallels to the idea of rational inattention [6, 7], as well as earlier work in neural coding [4, 17–19] and elsewhere in biology [20, 21].

While our navigation model is far too simple to quantitatively match data from any real organism, we can ask whether its qualitative features show up in the wild. Certainly many micro-organisms use strategies which alternate straight runs with sudden turns [22–24]. The transition from reverse to tumble being optimal (figures 1, 4A) could map to different bacteria which have evolved in different circumstances, or to adaptive behaviour by an individual. For example, *V. cholerae* performs a runreverse-flick strategy [3] when swimming fast, but only run-reverse when slow [25]. Intriguingly, the worm *C. elegans* uses a strategy qualitatively similar to our even steering μ(|θ|) when certain interneurons associated with navigation are disrupted [26]. For our second kind of discreteness, evidence suggests that flies adopt discrete turn saccades with stereotyped angles [27], and it would be interesting to explore how this phenomenon varies with the noise level.

For our stated problem, the optimal solution is to steer in the direction of θ=0, the red line in Figure 1. This could be interpreted as evidence for the high value of directional information. But it’s not entirely clear that we should directly compare the information rate of such steering strategies to the others. If a limited information rate is a proxy for sensor noise, then it is difficult to imagine a sensor giving directional and undirected information with equal ease. For instance with a bilateral pair of sensors, the difference will be a noisy directional measure, while the sum will be less-noisy but non-directional. The phase diagram of optimal strategies in this instance is unknown.

Aside from its biological relevance, our model is an unusual control theory problem in that it permits an exact solution for the steering rate μ(θ) for any information rate. The well-studied regime there is small deviations from θ=0, high information rate i, but our solution still holds in the opposite limit where the agent often makes full circles (as in figure 2E). We hope to present a proof that this strategy saturates a nonlinear performance bound soon [15].

It is a limitation of our model that we do not consider time-dependent or memory-based strategies, which are used by many real microorganisms. At the simplest level, bacteria don’t measure θ, but get a signal proportional to cos⁡θ from the derivative of attractant concentration along their path. This involves storing knowledge about the past for a time of order $1/D_{r}$ [14, 28, 29]. Many microorganisms swim in spirals in three dimensions [30]; this strategy could, using memory, gather information about spatial variations perpendicular to their track. In some situations *C. elegans* performs a weathervane motion [31], which could similarly leverage memory to measure spatial gradients. We plan to explore strategies which rely on memory in future work.

Finally, the mean speed up the gradient, $v/v_{0}$, isn’t the only relevant objective. If what’s being sensed is some localised food source, then there will be scenarios where the ability to loiter near to a point is also important [28], and scenarios where all that matters is being the first to arrive. For travelling groups of chemotactic bacteria [32], other objectives may be important for ensuring collective navigation of an isogenetic population. Perhaps these objectives are amenable to similar study.

More broadly, looking beyond questions of navigation, we believe that this work illustrates the utility of asking why biology chooses discrete solutions to continuous problems. Surely there are many other cases where evolution has arrived at a discrete strategy not because it was good enough, but because it was the best. And such cases are exciting because they can offer predictions for what should be seen under other conditions.

## Supplementary Material

Supplement 1

## Data Availability

Code which generates the figures is available here: github.com/mcabbott/ToSteerOrNot.jl
