# Supplementary material for: Discrete turn strategies emerge in information-limited navigation
Source: ArXiv. 2026 Jun 22:arXiv:2602.23324v2. Preprint. [Version 2] (PMC13321328)
Supplement: Supplement 1 [file NIHPP2602.23324v2-supplement-1.pdf]

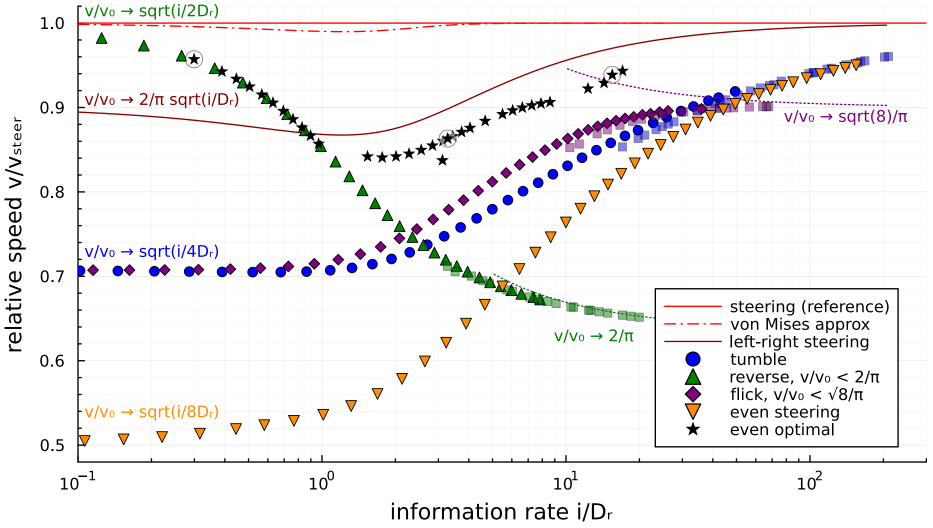

Figure S1: Performance of strategies for two-dimensional navigation, relative to steering. Shows most of the data in figure 1, but adds a lines for the exact left-right steering solution (dark red, section C.5), and numerical points for the flick solution (purple) and more optimal symmetric solutions $\lambda(|\Delta\theta|, \theta)$ (black stars). The three lines are strategies exploiting the sign of θ, with odd $\mu(\theta)$. If we ignore the black stars, then notice that three different even solutions are optimal in turn – reverse at low information rate, then flick, then tumble. As before, square plot points indicate the use of the ansatz $\lambda_{\mathrm{strong}}(\theta)$, while others solve for the whole function.

Appendix A. Additional figures

Figure 1 in the main text compares the performance of four strategies, and observes that steering (the red line) is fastest. Figure S1 shows the same data plotted relative to the steering solution, and adds several more strategies.

Figures S2 and S3 show some more numerical trajectories, first with the same parameters as figure 2E in the main text (all $i/D_r \approx 0.1$), and then with much higher information rates ($i/D_r \approx 5$). Observe particularly the steering solutions without the sign (rightmost panels) which make progress diagonally, and make occasional full turns. We make a sign choice in plotting these, as there is always an equivalent strategy, $-\mu(\theta)$, which goes diagonally the other way.

Appendix B. Analytic results for factorised discrete strategies

Here we derive some results for solutions whose jump rate is of the form $\lambda(\Delta\theta, \theta) = \lambda(\theta)q(\Delta\theta)$, where q is a probability distribution over target angles. We also assume that the target distribution is symmetric, such that $q(\Delta\theta) = q(-\Delta\theta)$. The tumble, reverse, and flick strategies are in this class.

At low information rates, we study small deviations from constant $\lambda(\theta)$, and are able to derive the $v \propto \sqrt{i}$ scaling law. We then show how to introduce a finite time-penalty per discrete action, and derive the effect on the $v \propto \sqrt{i}$ law. Finally, in Section B.3 we make an ansatz for steady-state $p(\theta)$ to be a von Mises distribution, which works well for the reverse strategy.

For this class of strategies, the Fokker-Planck equation reads

$$\frac{dp(\theta)}{dt} = -\lambda(\theta)p(\theta) + \int d\Delta\theta\, \lambda(\theta - \Delta\theta)p(\theta - \Delta\theta)q(\Delta\theta) + D_r p''(\theta), \tag{B1}$$

and the information rate is the same as (3)

$$i = \int d\theta\, p(\theta)\lambda(\theta) \log\left(\frac{\lambda(\theta)}{\langle\lambda\rangle}\right), \tag{B2}$$

where $\langle\lambda\rangle = \langle\lambda(\theta)\rangle_\theta = \int d\theta\, p(\theta)\lambda(\theta)$.

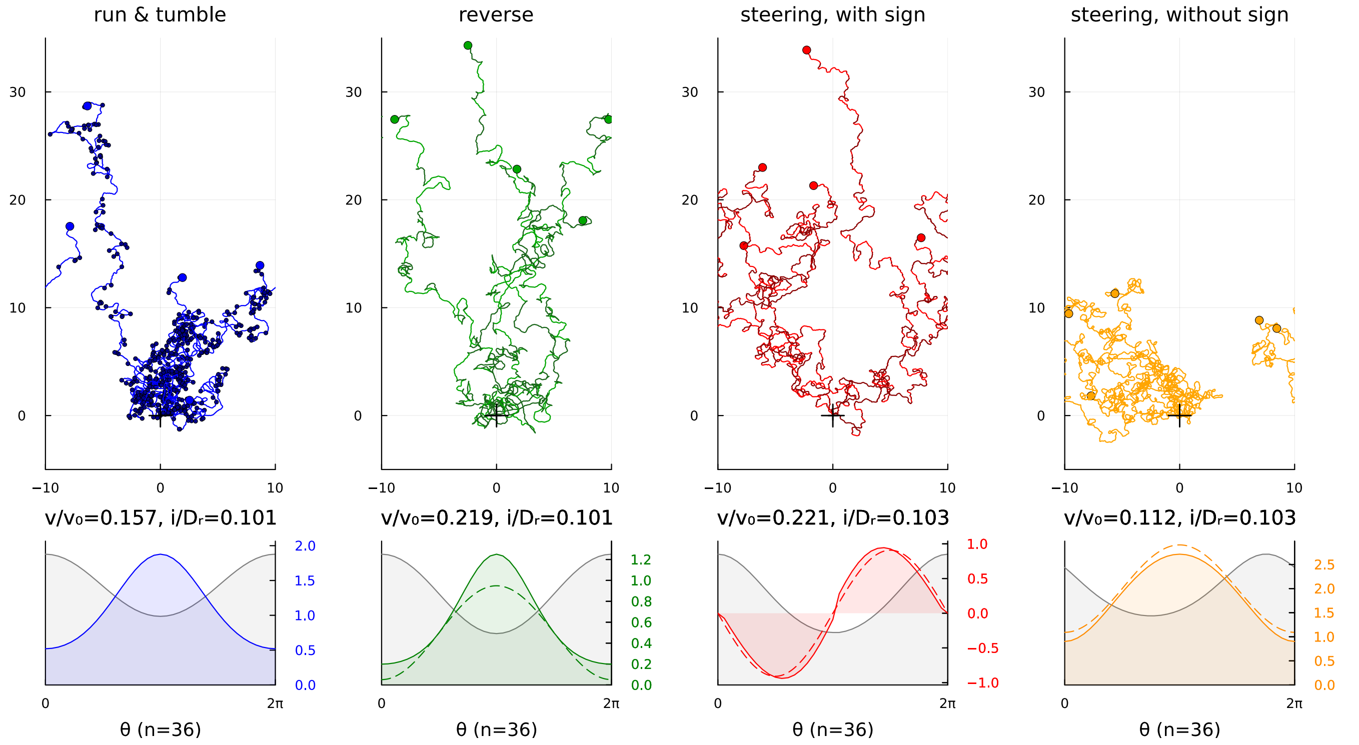

Figure S2: Sample trajectories for four strategies, all with the same information rate $i/D_r \approx 0.1$. Similar to as figure 2E, except showing five examples of each strategy. Each reversal changes the line colour between dark and light green. Steering changes between light and dark red according to the sign of $\mu(\theta)$. At low information rates, even steering $\mu(|\theta|)$ has $\langle\cos\theta\rangle_\theta \approx \pm\langle\sin\theta\rangle_\theta$, so the agent goes sideways as much as up the gradient. All plots are for time $0 < t < 100$, and wrapped to $-10 < x < 10$, in units $D_r = v_0 = 1$.

B.1 Solution at low information rate

At low information rate, we use a perturbative ansatz. We posit that there is some parameter ϵ, which vanishes as $\gamma \to \infty$, such that our strategy can be written as

$$\lambda(\theta) = \lambda_c(1 + \epsilon\lambda^{(1)}(\theta) + \epsilon^2\lambda^{(2)}(\theta) + \ldots), \tag{B3}$$

where the only dependency on γ is through ϵ and potentially λ_c. Since we have the freedom to choose λ_c and ϵ to set the scale, we can choose our first-order perturbation to satisfy

$$\int d\theta\, \lambda^{(1)}(\theta) = 0, \quad \frac{1}{2\pi}\int d\theta\, \lambda^{(1)}(\theta)^2 = 1. \tag{B4}$$

Intuitively, we allow for a non-zero rate as $\epsilon \to 0$ since all constant strategies use no information, but also have zero drift velocity. The second constraint ensures that the scale of the perturbation is fixed by ϵ.

We also expand the stationary density in ϵ:

$$p(\theta) = \frac{1}{2\pi}(1 + \epsilon p^{(1)}(\theta) + \epsilon^2 p^{(2)}(\theta) + \ldots). \tag{B5}$$

Due to normalization, we must have

$$\int d\theta\, p^{(k)}(\theta) = 0. \tag{B6}$$

Under these choices, the rest of the structure of the solution should arise from optimality. We allow the distribution $q(\Delta\theta)$ to be arbitrary, as long as it is symmetric.

Using our ansatz in the master equation and collecting the first-order terms in ϵ, we obtain

$$0 = \frac{D_r}{2\pi}\partial_\theta^2 p^{(1)}(\theta) - \frac{\lambda_c}{2\pi}[p^{(1)}(\theta) + \lambda^{(1)}(\theta)] + \frac{\lambda_c}{2\pi}\int d\theta'\, [p^{(1)}(\theta') + \lambda^{(1)}(\theta')]q(\theta - \theta'). \tag{B7}$$

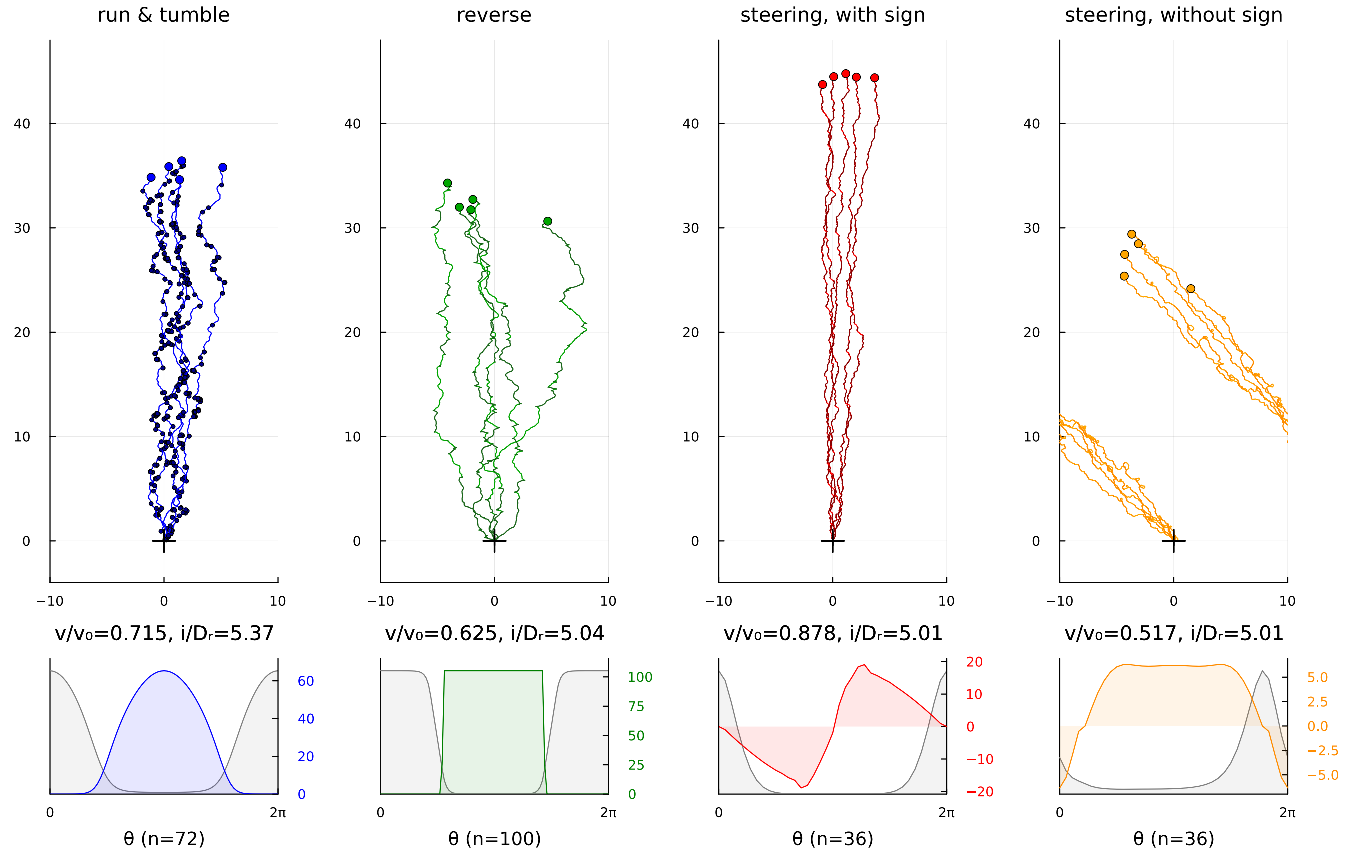

Figure S3: Sample trajectories for four strategies, with a higher information rate $i/D_r \approx 5$. All plots for time $0 < t < 50$. Compared to figure S2, these are half the duration, with 50x the information rate.

We now transform this equation to obtain its Fourier coefficients, using the following convention:

$$g_n = \frac{1}{2\pi} \int d\theta \, e^{-in\theta} g(\theta). \tag{B8}$$

With this, the Fourier modes of our first-order corrections satisfy

$$0 = -D_r n^2 p_n^{(1)} - \lambda_c [p_n^{(1)} + \lambda_n^{(1)}] + 2\pi \lambda_c [p_n^{(1)} + \lambda_n^{(1)}] q_n. \tag{B9}$$

Solving for $p_n^{(1)}$ we obtain

$$p_n^{(1)} = -\left(\frac{\lambda_c [1 - 2\pi q_n]/D_r}{n^2 + \lambda_c [1 - 2\pi q_n]/D_r} \right) \lambda_n^{(1)}. \tag{B10}$$

Therefore, our drift velocity can be written explicitly in terms of our strategy (λ, q):

$$\langle \cos(\theta) \rangle_\theta = \epsilon \frac{p_1^{(1)} + p_{-1}^{(1)}}{2} + \mathcal{O}(\epsilon^2) = -\frac{\epsilon}{2} \left(\frac{\lambda_c [1 - 2\pi q_1]/D_r}{1 + \lambda_c [1 - 2\pi q_1]/D_r} \right) [\lambda_1^{(1)} + \lambda_{-1}^{(1)}] + \mathcal{O}(\epsilon^2), \tag{B11}$$

where we used the symmetry of q to conclude that $q_1 = q_{-1} \in \mathbb{R}$.

Just like we can do a perturbative expansion of the drift velocity, we can do a perturbative expansion of the information rate. The leading order of this expansion will be ϵ^2, so we want to keep all terms up to this order. First, we compute the average

$$\langle \lambda \rangle = \frac{\lambda_c}{2\pi} \int d\theta \, [1 + \epsilon p^{(1)}(\theta) + \epsilon^2 p^{(2)}(\theta) + \ldots][1 + \epsilon \lambda^{(1)}(\theta) + \epsilon^2 \lambda^{(2)}(\theta) + \ldots]. \tag{B12}$$

We obtain

$$\langle\lambda\rangle = \lambda_c\left[1+\epsilon^2\lambda_0^{(2)}+\epsilon^2\frac{1}{2\pi}\int d\theta\, p^{(1)}(\theta)\lambda^{(1)}(\theta)+\mathcal{O}(\epsilon^3)\right]. \tag{B13}$$

Doing a perturbative expansion of the logarithm, this implies that

$$\log\left(\frac{\lambda_c}{\langle\lambda\rangle}\right) = -\epsilon^2\lambda_0^{(2)}-\epsilon^2\frac{1}{2\pi}\int d\theta\, p^{(1)}(\theta)\lambda^{(1)}(\theta)+\mathcal{O}(\epsilon^3). \tag{B14}$$

Similarly, we have that

$$\log\left(\frac{\lambda(\theta)}{\lambda_c}\right) = \epsilon\lambda^{(1)}(\theta)+\epsilon^2\lambda^{(2)}(\theta)-\frac{\epsilon^2}{2}\lambda^{(1)}(\theta)^2+\mathcal{O}(\epsilon^3). \tag{B15}$$

Grouping terms together, we obtain

$$\log\left(\frac{\lambda(\theta)}{\langle\lambda\rangle}\right) = \epsilon\lambda^{(1)}(\theta)+\epsilon^2\left[\lambda^{(2)}(\theta)-\lambda_0^{(2)}-\frac{1}{2}\lambda^{(1)}(\theta)^2-\frac{1}{2\pi}\int d\theta'\, p^{(1)}(\theta')\lambda^{(1)}(\theta')\right]+\mathcal{O}(\epsilon^3). \tag{B16}$$

The expansion for $p(\theta)\lambda(\theta)$ is

$$p(\theta)\lambda(\theta) = \frac{\lambda_c}{2\pi}\left[1+\epsilon(p^{(1)}(\theta)+\lambda^{(1)}(\theta))+\mathcal{O}(\epsilon^2)\right]. \tag{B17}$$

These two expansions are sufficient to obtain all the terms to order ϵ^2.

Let us now decompose the information rate into two terms: $i = i_1 + i_2 + \mathcal{O}(\epsilon^3)$, given by

$$\begin{aligned} i_1 &= \epsilon^2\frac{\lambda_c}{2\pi}\int d\theta\, [p^{(1)}(\theta)+\lambda^{(1)}(\theta)]\lambda^{(1)}(\theta), \\ i_2 &= \epsilon^2\frac{\lambda_c}{2\pi}\int d\theta\, \left[\lambda^{(2)}(\theta)-\lambda_0^{(2)}-\frac{1}{2}\lambda^{(1)}(\theta)^2-\frac{1}{2\pi}\int d\theta'\, p^{(1)}(\theta')\lambda^{(1)}(\theta')\right]. \end{aligned} \tag{B18}$$

In i_2, we can easily see that the first two terms cancel, so we obtain

$$i_2 = -\epsilon^2\frac{\lambda_c}{2\pi}\int d\theta\, \left[p^{(1)}(\theta)\lambda^{(1)}(\theta)+\frac{1}{2}\lambda^{(1)}(\theta)^2\right]. \tag{B19}$$

Bringing these terms together, we obtain the leading-order term for the information rate:

$$i = \frac{\epsilon^2\lambda_c}{4\pi}\int d\theta\, \lambda^{(1)}(\theta)^2+\mathcal{O}(\epsilon^3) = \frac{1}{2}\epsilon^2\lambda_c+\mathcal{O}(\epsilon^3), \tag{B20}$$

where the last equality comes from the choice of scale we made in our expansion.

We can now state the optimization problem for the leading-order terms in drift velocity and information rate:

$$\max_{\lambda^{(1)},\lambda_c,\epsilon}\left\{\underbrace{-\frac{\epsilon}{2}\left(\frac{\lambda_c[1-2\pi q_1]/D_r}{1+\lambda_c[1-2\pi q_1]/D_r}\right)[\lambda_1^{(1)}+\lambda_{-1}^{(1)}]}_{v/v_0}-\gamma\underbrace{\frac{1}{2}\epsilon^2\lambda_c}_{i}\right\}. \tag{B21}$$

Note that we allow ϵ to be chosen optimally. Our solution is consistent if the optimal epsilon vanishes as $\gamma\to\infty$. Additionally, we keep our q strategy fixed for now. First, note that the information rate does not depend on the structure of the $\lambda^{(1)}$ modes. Therefore, it is optimal to just keep the $n=\pm1$ modes and choose them to be $-1/\sqrt{2}$, such that $\lambda^1(\theta) = -\sqrt{2}\cos(\theta)$. The optimization problem, then, becomes

$$\max_{\lambda_c,\epsilon}\left\{\frac{\epsilon}{\sqrt{2}}\left(\frac{\lambda_c[1-2\pi q_1]/D_r}{1+\lambda_c[1-2\pi q_1]/D_r}\right)-\gamma\frac{1}{2}\epsilon^2\lambda_c\right\}. \tag{B22}$$

Solving for the optimal ϵ and λ_c yields

$$\begin{aligned} \epsilon^* &= \frac{1-2\pi q_1}{2\sqrt{2}\gamma D_r}, \\ \lambda_c^* &= \frac{D_r}{1-2\pi q_1}. \end{aligned} \tag{B23}$$

This justifies our initial choice of the perturbative ansatz, since ϵ^* scales as $1/\gamma$ and λ_c^* is independent of γ.

Under the optimal strategy, we can find a relationship between the drift velocity and the information rate, which yields our low-information Pareto frontier:

$$\frac{v}{v_0} = \left(\frac{i[1-2\pi q_1]}{4D_r}\right)^{1/2}. \tag{B24}$$

We can see what this frontier is for some of the strategies we analyze in the main text. For run and tumble, we have $q(\Delta\theta) = 1/2\pi$, so $q_1 = 0$. Therefore, its Pareto frontier scales as

$$\left(\frac{v}{v_0}\right)_{\text{tumbles}} = \left(\frac{i}{4D_r}\right)^{1/2}. \tag{B25}$$

The target distribution for the reversing strategy is $q(\Delta\theta) = \delta(\Delta\theta - \pi)$. This means $q_1 = -1/2\pi$, so the Pareto frontier is

$$\left(\frac{v}{v_0}\right)_{\text{reverse}} = \left(\frac{i}{2D_r}\right)^{1/2}. \tag{B26}$$

In fact, reversing achieves the highest possible drift velocity in this regime. For any symmetric strategy q, we have that

$$q_1 = \frac{1}{2\pi}\int_0^{2\pi} d\Delta\theta\, e^{-i\Delta\theta} q(\Delta\theta) = \frac{1}{2\pi}\int_0^{2\pi} d\Delta\theta\, \cos(\Delta\theta) q(\Delta\theta) = \frac{1}{2\pi}\langle\cos(\Delta\theta)\rangle_q. \tag{B27}$$

Since $\cos(\Delta\theta) \geq -1$, we must have

$$q_1 \geq -\frac{1}{2\pi}. \tag{B28}$$

Since the reversing strategy saturates this bound, and the scaling of the frontier only depends on q_1, we conclude that it is the optimal low-information strategy.

In addition to the information rate, we can find the leading-order behavior of the jump rate. In terms of λ^* and ϵ^*, this is

$$\lambda(\theta) \approx \lambda_c^*\left[1 - \sqrt{2}\epsilon^* \cos(\theta)\right]. \tag{B29}$$

Using our solution and writing these in terms of i, we obtain

$$\lambda(\theta) \approx \frac{D_r}{1-2\pi q_1}\left[1 - 2\left(\frac{i}{D_r}\right)^{1/2}(1-2\pi q_1)^{1/2}\cos(\theta)\right]. \tag{B30}$$

B.2 Time penalty for tumbles or other actions

So far we have considered instantaneous changes of heading, but any real organisms takes a finite amount of time to turn. For example, tumbles take up order 10% of an *E. coli*'s time. To build this into our framework, we now introduce a time penalty τ for each tumble, or other action. We write $\bar{p}_\tau(t)$ for the proportion of time spent tumbling, and $\bar{p}(\theta, t)$ for the rest of the time. These obey

$$\bar{p}_\tau(t) = \int_{t-\tau}^{t} dt' \int d\theta\, \lambda(\theta)\, \bar{p}(\theta, t'), \qquad \bar{p}_\tau(t) + \int d\theta\, \bar{p}(\theta, t) = 1. \tag{B31}$$

The Fokker-Planck equation for $d\bar{p}(\theta,t)/dt$ takes the same form as above, except that it needs a time delay in the source term: $\int d\Delta\theta\, \lambda(\theta - \Delta\theta)\, \bar{p}(\theta - \Delta\theta, t - \tau) q(\Delta\theta)$. This linear equation has steady-state solution $\bar{p}(\theta, t) = \eta\, p(\theta)$, where $p(\theta)$ is the normalized density solving the original ($\tau = 0$) equation above. Solving, we get

$$\bar{p}_\tau(t) = 1 - \eta, \qquad \eta = \frac{1}{1 + \tau\, \langle\lambda(\theta)\rangle_\theta}, \tag{B32}$$

where the expectation value is still with respect to $p(\theta)$, i.e. $\langle\lambda(\theta)\rangle_\theta = \int d\theta\, p(\theta)\lambda(\theta)$. The up-gradient relative speed averages over only the time not spent tumbling:

$$v/v_0 = \int d\theta\, \bar{p}(\theta,t)\cos\theta = \eta\, \langle\cos\theta\rangle_\theta\,. \tag{B33}$$

Therefore, our optimization problem now reads:

$$\max_{\lambda,q} \frac{\langle\cos\theta\rangle_\theta}{1+\tau\,\langle\lambda(\theta)\rangle_\theta} - \gamma\, i. \tag{B34}$$

In the low information regime, we can use the perturbative expansions in the previous section. From Eq. (B13), we have that the corrections to $\langle\lambda\rangle$ are of second order in ϵ. Therefore, following the procedure in the previous section, we arrive at the leading-order optimization problem for a fixed q:

$$\max_{\lambda_c,\epsilon}\left\{\frac{\epsilon}{\sqrt{2}(1+\tau\lambda_c)}\left(\frac{\lambda_c[1-2\pi q_1]/D_r}{1+\lambda_c[1-2\pi q_1]/D_r}\right) - \gamma\frac{1}{2}\epsilon^2\lambda_c\right\}. \tag{B35}$$

Recall that q_1 is the first Fourier mode of $q(\Delta\theta)$. Solving the problem gives the frontier

$$\frac{v}{v_0} = \left(\frac{i[1-2\pi q_1]}{4D_r}\right)^{1/2} U\left(\frac{D_r\tau}{1-2\pi q_1}\right), \tag{B36}$$

where U is a universal function given by

$$U(x) := \frac{2\sqrt{\phi(x)}}{(1+x\phi(x))(1+\phi(x))}, \quad \phi(x) := \frac{-(1+x)+\sqrt{(1+x)^2+12x}}{6x}. \tag{B37}$$

Importantly, U is decreasing and satisfies $U(0) = 1$ and $U(x\to\infty)\to 0$. For intuition, it useful to note that U satisfies

$$\left(\frac{1}{2+x}\right)^{1/2} \le U(x) \le \left(\frac{1}{1+x}\right)^{1/2}. \tag{B38}$$

We can evaluate the value of the time delay τ for which tumbling and reversing perform worse than even steering at low information. For tumbles $q_1 = 0$, so the critical τ is determined by

$$U(D_r\tau_{\text{tumbles}}) = \frac{1}{\sqrt{2}} \quad\Longrightarrow\quad D_r\tau_{\text{tumbles}} \approx 0.694. \tag{B39}$$

For reversing, we have $q_1 = -1/2\pi$. Therefore, the critical τ is determined by

$$U\left(\frac{D_r\tau_{\text{reverse}}}{2}\right) = \frac{1}{2} \quad\Longrightarrow\quad D_r\tau_{\text{reverse}} \approx 4.907. \tag{B40}$$

B.3 Parametric ansatz for reverse strategies

For a given strategy, we can parametrize a form of the stationary distribution that seems consistent with the numerical solutions. In particular, we will characterize the distributions in terms of a width parameter κ, and denote them with $p_\kappa(\theta)$. Our optimization problem, then, is

$$\max_{\kappa,\lambda}\left\{\langle\cos(\theta)\rangle_\theta - \gamma i\right\}, \tag{B41}$$

subject to the condition that λ generates p_κ as the stationary distribution. Conditional on κ, the drift velocity $\langle\cos(\theta)\rangle_\theta$ is only determined by p_κ. Therefore, we can solve the optimization process in two steps. First, define

$$\lambda_\kappa := \operatorname*{argmin}_\lambda i. \tag{B42}$$

That is, first we solve for the jump rate that minimizes the information rate subject to the master-equation constraint. Then we can perform the maximization over κ for a given γ. However, if the problem is sufficiently nice, we only need to solve the problem (min λ) and the Pareto frontier will be traced out by varying κ.

The stationarity condition for reversing takes the form

$$D_r p''(\theta) - p(\theta)\lambda(\theta) + p(\theta+\pi)\lambda(\theta+\pi) = 0. \tag{B43}$$

This gives an interesting constraint on our stationary distribution:

$$p''(\theta) = -p''(\theta+\pi). \tag{B44}$$

Knowing this, and looking at the numerical results, we propose the following ansatz for the stationary distribution:

$$p_\kappa(\theta) = \frac{1}{\pi}\Phi(\kappa\cos(\theta)), \tag{B45}$$

where Φ is a smooth CDF, which satisfies $\Phi(-x) = 1 - \Phi(x)$. This distribution satisfies the anti-symmetry constraint and smoothly interpolates between a uniform distribution and a uniform distribution on the interval $[-\pi/2, \pi/2]$.

We want to optimize for the jump rate that minimizes the information rate. We can write the information rate in terms of the jump rate on the interval $[-\pi/2, \pi/2]$:

$$i = \int_{-\pi/2}^{\pi/2} d\theta \left[p(\theta)\lambda(\theta)\log\left(\frac{\lambda(\theta)}{\langle\lambda\rangle}\right) + p(\theta+\pi)\lambda(\theta+\pi)\log\left(\frac{\lambda(\theta+\pi)}{\langle\lambda\rangle}\right)\right]. \tag{B46}$$

This allows us to explicitly incorporate the constraint, since $\lambda(\theta)$ and $\lambda(\theta+\pi)$ cannot be independently varied. Using our constraint, we can write this as

$$i = \int_{-\pi/2}^{\pi/2} d\theta \left[p(\theta)\lambda(\theta)\log\left(\frac{\lambda(\theta)}{\langle\lambda\rangle}\right) + [p(\theta)\lambda(\theta) - D_r p''(\theta)]\log\left(\frac{p(\theta)\lambda(\theta) - D_r p''(\theta)}{p(\theta+\pi)\langle\lambda\rangle}\right)\right]. \tag{B47}$$

Additionally, we can write the average jumping rate as

$$\langle\lambda\rangle = \int_{-\pi/2}^{\pi/2} d\theta\, [p(\theta)\lambda(\theta) + p(\theta+\pi)\lambda(\theta+\pi)]. \tag{B48}$$

With the constraint, this becomes

$$\langle\lambda\rangle = \int_{-\pi/2}^{\pi/2} d\theta\, [2p(\theta)\lambda(\theta) - D_r p''(\theta)]. \tag{B49}$$

Simplifying:

$$\langle\lambda\rangle = 2\int_{-\pi/2}^{\pi/2} d\theta\, p(\theta)\lambda(\theta) - D_r[p'(\pi/2) - p'(-\pi/2)]. \tag{B50}$$

This gives us our first functional derivative:

$$\frac{\delta\langle\lambda\rangle}{\delta\lambda(\theta)} = 2p(\theta). \tag{B51}$$

With this setup, we can write our first-order condition as

$$\frac{\delta i}{\delta\lambda(\theta)} = 0. \tag{B52}$$

Taking this variation explicitly yields

$$\begin{aligned}\frac{\delta i}{\delta\lambda(\theta)} =& p(\theta)\log\left(\frac{\lambda(\theta)}{\langle\lambda\rangle}\right) + p(\theta)\log\left(\frac{p(\theta)\lambda(\theta) - D_r p''(\theta)}{p(\theta+\pi)\langle\lambda\rangle}\right)\\ &+ 2p(\theta) - \int_{-\pi/2}^{\pi/2} d\theta' \left[\frac{p(\theta')\lambda(\theta')}{\langle\lambda\rangle} + \frac{p(\theta')\lambda(\theta') - D_r p''(\theta')}{\langle\lambda\rangle}\right]\frac{\delta\langle\lambda\rangle}{\delta\lambda(\theta)}.\end{aligned} \tag{B53}$$

Note that this last integral evaluates to $2p(\theta)$. Therefore, we can simplify the FOC to

$$\log\left(\frac{\lambda(\theta)}{\langle\lambda\rangle}\right)+\log\left(\frac{p(\theta)\lambda(\theta)-D_r p''(\theta)}{p(\theta+\pi)\langle\lambda\rangle}\right)=0. \tag{B54}$$

This implies that

$$\lambda(\theta)\lambda(\theta+\pi)=\langle\lambda\rangle^2. \tag{B55}$$

Note that this result holds regardless of the parametric ansatz.

This result gets us most of the way to characterizing the solution. For a given κ, let $\tilde{\lambda}_\kappa(\theta):=\lambda_\kappa(\theta)/\langle\lambda_\kappa\rangle$. Then our optimality condition is

$$\tilde{\lambda}_\kappa(\theta)\tilde{\lambda}_\kappa(\theta+\pi)=1. \tag{B56}$$

Multiplying by $p(\theta)p(\theta+\pi)$ and using our constraint, we obtain

$$p_\kappa(\theta)\tilde{\lambda}_\kappa(\theta)\left[p_\kappa(\theta)\tilde{\lambda}_\kappa(\theta)-\frac{D_r}{\langle\lambda_\kappa\rangle}p''_\kappa(\theta)\right]=p_\kappa(\theta)p_\kappa(\theta+\pi). \tag{B57}$$

Another nice property of our ansatz is that $p_\kappa(\theta+\pi)=\frac{1}{\pi}-p_\kappa(\theta)$. Therefore, we obtain

$$p_\kappa(\theta)\tilde{\lambda}_\kappa(\theta)\left[p_\kappa(\theta)\tilde{\lambda}_\kappa(\theta)-\frac{D_r}{\langle\lambda_\kappa\rangle}p''_\kappa(\theta)\right]=p_\kappa(\theta)\left[\frac{1}{\pi}-p_\kappa(\theta)\right]. \tag{B58}$$

We can solve for $p_\kappa(\theta)\tilde{\lambda}_\kappa(\theta)$:

$$p_\kappa(\theta)\tilde{\lambda}_\kappa(\theta)=\frac{D_r}{2\langle\lambda_\kappa\rangle}p''_\kappa(\theta)+\left(\frac{D_r^2}{4\langle\lambda_\kappa\rangle^2}p''_\kappa(\theta)^2+p_\kappa(\theta)\left[\frac{1}{\pi}-p_\kappa(\theta)\right]\right)^{1/2}. \tag{B59}$$

Additionally, note that by definition

$$\int d\theta\, p_\kappa(\theta)\tilde{\lambda}_\kappa(\theta)=1. \tag{B60}$$

Therefore, $\langle\lambda_\kappa\rangle$ must satisfy the fixed-point equation

$$\langle\lambda_\kappa\rangle=\int d\theta\left(\frac{D_r^2}{4}p''_\kappa(\theta)^2+p_\kappa(\theta)\left[\frac{1}{\pi}-p_\kappa(\theta)\right]\langle\lambda_\kappa\rangle^2\right)^{1/2}. \tag{B61}$$

Once $\langle\lambda_\kappa\rangle$ is determined, the optimal jump rate is

$$\lambda_\kappa(\theta)=\frac{1}{p_\kappa(\theta)}\left[\frac{D_r}{2}p''_\kappa(\theta)+\left(\frac{D_r^2}{4}p''_\kappa(\theta)^2+p_\kappa(\theta)\left[\frac{1}{\pi}-p_\kappa(\theta)\right]\langle\lambda_\kappa\rangle^2\right)^{1/2}\right]. \tag{B62}$$

For a given κ, this gives us a routine to find a point on the estimated frontier, since we can evaluate the drift velocity and information rate using $p_\kappa(\theta)$ and $\langle\lambda_\kappa\rangle$.

Appendix C. Analytic results for continuous steering

Here we derive analytic results for the strategies when only continuous steering $\mu(\theta)$ is used, with no discrete jumps. First, we derive the information rate (3); see also Section E.2 below for a derivation in d dimensions.

Then we find some optimal solutions, starting with most general version where the agent can measure the full heading θ, which we call signed information. We also consider a variant where the agent has access only to the sign, left or right, for which we find a much simpler analytic solution. Finally, we treat the case of steering without the sign, where we have an analytic solution only at low information rates.

C.1 Steering and diffusion as turns $\Delta\theta = \pm\alpha$

We can write the controlled update for θ for steering and diffusion in short time Δt as follows:

$$\Delta\theta = \mu(\theta)\Delta t + \sqrt{2D_c\,\Delta t}\,\eta, \qquad \eta \sim \mathcal{N}(0,1). \tag{C1}$$

Averaging over the distribution $p(\Delta\theta|\theta)$, the mean change is $\langle\Delta\theta\rangle_{\Delta\theta\sim p(\Delta\theta|\theta)} = \mu(\theta)\Delta t$, and the variance is $\big\langle(\Delta\theta - \mu(\theta)\Delta t)^2\big\rangle_{\Delta\theta\sim p(\Delta\theta|\theta)} = 2D_c\Delta t$. We can reproduce these with small finite jumps of $\Delta\theta = \pm\alpha$, with rates $\beta_\pm(\theta)$:

$$\begin{aligned} p(\pm\alpha|\theta) &= \beta_\pm(\theta)\Delta t \\ p(0|\theta) &= 1 - \sum_\pm \beta_\pm(\theta)\Delta t. \end{aligned} \tag{C2}$$

Solving, we get

$$\beta_\pm(\theta) = \frac{D_c}{\alpha^2} \pm \frac{\mu(\theta)}{2\alpha} + \mathcal{O}(\Delta t). \tag{C3}$$

Here we must assume $D_c > \alpha\,|\mu(\theta)|/2 > 0$.

Now we can plug these probabilities into the mutual information:

$$I(\Theta;\Delta\Theta) = \int d\theta \sum_\pm p(\pm\alpha|\theta)p(\theta)\log\frac{p(\pm\alpha|\theta)}{p(\pm\alpha)} + \mathcal{O}(\Delta t^2) \tag{C4}$$

where the denominator is

$$p(\pm\alpha) = \int d\theta'\, p(\pm\alpha|\theta')p(\theta') = \left[\frac{D_c}{\alpha^2} \pm \frac{\langle\mu\rangle}{2\alpha}\right]\Delta t + \mathcal{O}(\Delta t^2). \tag{C5}$$

Then expanding at small α we get

$$I(\Theta;\Delta\Theta) = \int d\theta\, p(\theta)\sum_\pm\left[\frac{D_c}{\alpha^2} \pm \frac{\mu(\theta)}{2\alpha}\right]\log\frac{\frac{D_c}{\alpha^2} \pm \frac{\mu(\theta)}{2\alpha}}{\frac{D_c}{\alpha^2} \pm \frac{\langle\mu\rangle}{2\alpha}} + \dots \tag{C6}$$

$$= \Delta t\int d\theta\, p(\theta)\frac{[\mu(\theta) - \langle\mu\rangle]^2}{4D_c} + \mathcal{O}(\alpha, \Delta t^2) \tag{C7}$$

which is the desired formula, equation (4) in the main text. The formula, for tumbles, (3), can be derived in much the same way, taking $p(\text{tumble}|\theta) = \lambda(\theta)\Delta t$ and expanding in Δt like (C4).

Notice that the same steering rate $\mu(\theta)$ can be encoded as turns by a larger or smaller angle α, with larger α corresponding to smaller Poisson rate $\beta_\pm$ from (C3). This freedom is illustrated in figure S4A. When solving numerically for the optimal $\lambda(\Delta\theta,\theta)$, the same freedom gives a nearly-flat direction in the parameter space. This means that the performance (speed and information, figure 1) converges, while the exact rates do not. Figure S4B shows what we see but different solver choices will change things. ??

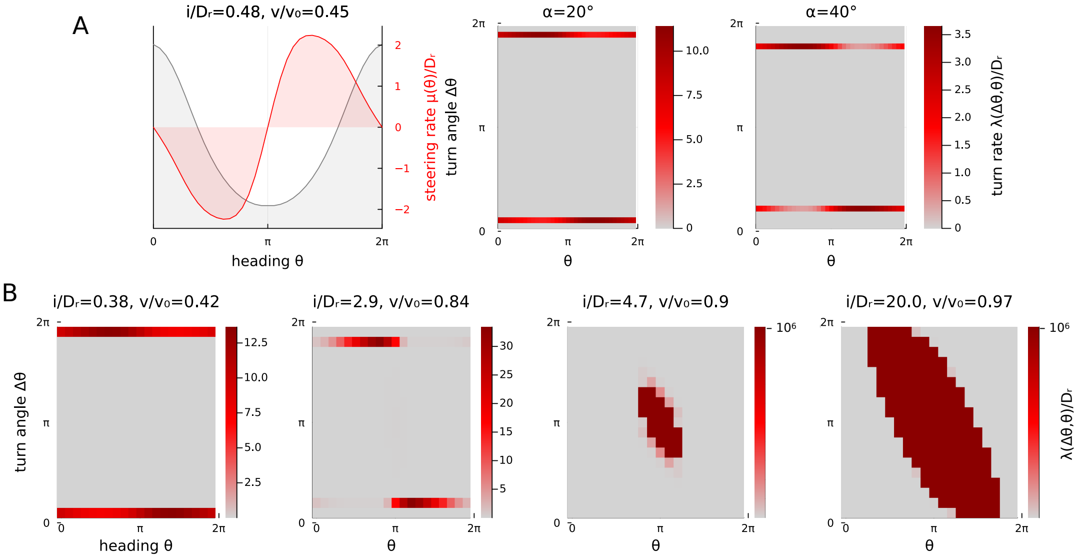

Figure S4: Numerical steering solutions. (A) Any steering rate $\mu(\theta)$ can be translated to a turn rate $\lambda(\Delta\theta, \theta)$ using angles $\Delta\theta = \pm\alpha$, and here we show the same solution translated to two different choices of α: either small-angle turns at a high rate, or larger-angle turns at a lower rate. (B) Some numerical solutions finding the unconstrained rate $\lambda(\Delta\theta, \theta)$ directly. These are among the solutions plotted on as red diamonds on figure 1. We believe that their performance has converged, but the exact rates $\lambda(\Delta\theta, \theta)$ shown here have not, and depend on details of the numerical algorithm used. This is because of the nearly flat direction in parameter space illustrated in panel A. At low information rates, the solution shown uses the smallest turns possible, i.e. sets α to the discretisation scale. But at high information rates, some solutions use larger angles, eventually pushing on the constraint $\lambda(\Delta\theta, \theta) \leq 10^6$ imposed on the solver.

C.2 Exact solution for steering $\mu(\theta)$

When $\mu(\theta)$ has full freedom, we expect by symmetry that there will be no net rotational flux of the heading. In this case we can integrate the Fokker-Planck equation after setting the steady-state condition $\partial_t p = 0$, to obtain a Boltzmann-like solution of the form

$$p(\theta) = \frac{1}{Z} \exp\left(\frac{1}{D_r + D_c} \int \mu(\theta') d\theta' \right), \tag{C8}$$

for partition function Z defined to ensure normalization. It follows then that the steering force can be written as a function of the probability distribution as

$$\mu(\theta) = (D_r + D_c)\partial_\theta \log p(\theta). \tag{C9}$$

This allows us to write the information rate as a function only of $p(\theta)$, as

$$i = \frac{1}{4D_c} \mathrm{Var}(\mu) \tag{C10a}$$

$$= \frac{(D_r + D_c)^2}{4D_c} \int_0^{2\pi} p(\theta) \left[\partial_\theta \log p(\theta)\right]^2 d\theta. \tag{C10b}$$

We can then write the objective function as a functional integral,

$$\mathcal{L} = \int_0^{2\pi} d\theta \underbrace{\left[p(\theta)\cos\theta - \frac{\gamma(D_r + D_c)^2}{4D_c} p(\theta) \left[\partial_\theta \log p(\theta)\right]^2 + \alpha \left[p(\theta) - \frac{1}{2\pi} \right] \right]}_{f(\theta, p, p')}. \tag{C11}$$

Here α is a Lagrange multiplier ensuring normalization of $p(\theta)$. Assuming that $\mu(\theta)$ has sufficient freedom to tune $p(\theta)$ as needed for any D_c, we can optimize $\mathcal{L}$ with respect to D_c at constant $p(\theta)$, which yields $D_c = D_r$. We then

extremize $\mathcal{L}$ by deriving the Euler-Lagrange equation, obtaining the following differential equation which $p(\theta)$ must solve:

$$0 = \alpha + \cos\theta + \gamma D_r \left(\frac{p'(\theta)}{p(\theta)}\right)^2 + 2\gamma D_r \frac{d}{d\theta}\left(\frac{p'(\theta)}{p(\theta)}\right). \tag{C12}$$

This differential equation falls within the Mathieu family, and can thus be solved exactly in closed form using special functions; after enforcing normalization and rotational symmetry this yields

$$p(\theta) = \frac{1}{2\pi}\mathrm{ce}_0\left(\theta/2, q\right)^2. \tag{C13}$$

Here ce_0 is the zeroth order Mathieu C function of the first kind [33], and $q = -1/(2D_r\gamma)$ is a dimensionless parameter. This distribution is normalized and periodic on $\theta \in [0, 2\pi]$, with a peak at $\theta = 0$ for $\gamma > 0$. We use the standard convention that $\int_0^\pi \mathrm{ce}_0(x,q)^2 \mathrm{d}x = \pi$. We will call $p(\theta) = \frac{1}{2\pi}\mathrm{ce}_0\left(\theta/2, q\right)^2$ the Mathieu distribution. This distribution has previously been studied as the solution to some problems in quantum mechanics [34, 35]. The corresponding steering force is given by

$$\mu(\theta) = 4D_r \frac{\partial_\theta \mathrm{ce}_0\left(\theta/2, q\right)}{\mathrm{ce}_0\left(\theta/2, q\right)}, \tag{C14}$$

where ∂_θ denotes a partial derivative with respect to θ.

C.3 Pareto Frontier

For this steering strategy it is possible to evaluate both the average velocity and information rate in closed form using the Mathieu characteristic function $a_0(q)$ and its derivative $a_0'(q)$. To do so, we first note that the Mathieu function $y(x) = \mathrm{ce}_0(x, q)$ satisfies the ODE

$$\underbrace{\left[-\frac{\mathrm{d}^2}{\mathrm{d}x^2} + 2q\cos(2x)\right]}_{=H(q)} y = a_0(q)y, \tag{C15}$$

where we have defined the operator $H(q)$, which we note is self-adjoint as a result of periodic boundary conditions. Taking a partial derivative with respect to q, multiplying both sides by y, and integrating over x from 0 to π, we obtain

$$\int_0^\pi y\frac{\partial H}{\partial q}y\mathrm{d}x + \int_0^\pi yH(q)\frac{\partial y}{\partial q}\mathrm{d}x = a_0'(q)\int_0^\pi y^2\mathrm{d}x + a_0(q)\int_0^\pi y\frac{\partial y}{\partial q}\mathrm{d}x. \tag{C16}$$

Using the self-adjoint nature of H, we can write

$$\int_0^\pi yH(q)\frac{\partial y}{\partial q}\mathrm{d}x = \int_0^\pi H(q)y\frac{\partial y}{\partial q}\mathrm{d}x \tag{C17a}$$

$$= a_0(q)\int_0^\pi y\frac{\partial y}{\partial q}\mathrm{d}x. \tag{C17b}$$

This simplifies Eq. (C16) to

$$\int_0^\pi y\frac{\partial H}{\partial q}y\mathrm{d}x = a_0'(q)\int_0^\pi y^2\mathrm{d}x. \tag{C18}$$

Noting now that $\partial H/\partial q = 2\cos(2x)$, we can simplify and rearrange this equation to yield

$$\frac{1}{2}a_0'(q) = \frac{\int_0^\pi \cos(2x)y^2\mathrm{d}x}{\int_0^\pi y^2\mathrm{d}x}. \tag{C19}$$

Since our solution $p(\theta) = y(\theta/2)^2/\pi$, the right hand side of this equation is exactly $\langle\cos\theta\rangle$, so that we have

$$\langle\cos\theta\rangle = \frac{1}{2}a_0'(q). \tag{C20}$$

We then turn to evaluate the information rate i, which we can write in terms of $y(x)$ as

$$i/D_r = \int_0^{2\pi} p(\theta) \left[\partial_\theta \ln p(\theta)\right]^2 \mathrm{d}\theta \tag{C21a}$$

$$= \frac{\int_0^\pi (y'(x))^2 \mathrm{d}x}{\int_0^\pi y(x)^2 \mathrm{d}x}. \tag{C21b}$$

To evaluate this expression, we return to our original ODE for y, multiply both sides by y, and integrate from $x = 0$ to π to obtain

$$\int_0^\pi yy'' \mathrm{d}x + a_0(q) \int_0^\pi y^2 \mathrm{d}x = 2q \int_0^\pi \cos(2x) y^2 \mathrm{d}x. \tag{C22}$$

Using our result (C19), we can simplify this to

$$qa_0'(q) - a_0(q) = \frac{\int_0^\pi yy'' \mathrm{d}x}{\int_0^\pi y^2 \mathrm{d}x}. \tag{C23}$$

Using integration by parts to show $\int yy'' = -\int y'^2$, we can then relate the right-hand side to the information rate, for which we obtain

$$i/D_r = a_0(q) - qa_0'(q). \tag{C24}$$

We thus have a parametric form of the Pareto frontier that can easily be evaluated numerically:

$$v/v_0 = \frac{1}{2} a_0'(q), \tag{C25a}$$

$$i/D_r = a_0(q) - qa_0'(q). \tag{C25b}$$

C.4 Approximations to the solution for steering $\mu(\theta)$

As has been noted previously [34, 35], the Mathieu distribution is well-approximated by a von-Mises distribution for both large and small q, which correspond to the low and high information limits respectively. The von-Mises distribution takes the form

$$p(\theta) = \frac{1}{2\pi I_0(\kappa)} \exp\left[\kappa \cos(\theta)\right], \tag{C26}$$

with shape parameter κ. $I_0(\kappa)$ is the 0'th order modified Bessel function of the first kind.

As $q \to 0$ (low information) the Mathieu distribution is approximated by a von-Mises distribution with $\kappa = -q$, which leads to a steering strategy

$$\mu(\theta) \approx -\frac{1}{\gamma} \sin\theta. \tag{C27}$$

With this steering strategy we obtain the scaling

$$v/v_0 \approx \sqrt{\frac{i/D_r}{2}}. \tag{C28}$$

Conversely, as $q \to -\infty$ (high information), the Mathieu distribution is approximated by a von-Mises distribution with $\kappa = \sqrt{-q}$, leading to a steering strategy

$$\mu(\theta) \approx -\sqrt{\frac{2D_r}{\gamma}} \sin\theta, \tag{C29}$$

and velocity-information scaling

$$v/v_0 \approx 1 - \frac{1}{2i/D_r}. \tag{C30}$$

More generally, we find that the von-Mises ansatz for $p(\theta)$ is a remarkably good approximation to the true optimal solution even at intermediate information rates. This functional form allows us to derive an implicit approximation for the Pareto frontier,

$$\frac{v}{v_0} = \frac{I_1\left(\frac{i/D_\mathrm{r}}{v/v_0}\right)}{I_0\left(\frac{i/D_\mathrm{r}}{v/v_0}\right)}, \tag{C31}$$

with I_0 and I_1 the 0'th and first order modified Bessel function of the first kind

C.5 Sign-only steering $\mu_{\text{left-right}}(\theta)$

For steering, perhaps the simplest solution uses only the sign of θ, adopting a fixed steering rate left or right as required. This could be the case if, for example, the steering force is constrained to only take a single magnitude $|M|$, or if only the sign of θ can be measured. Using only the sign of θ, the control strategy must take on the following odd functional form:

$$\mu_{\text{left-right}}(\theta) = \begin{cases} -M, & 0 < \theta < \pi \\ M, & \text{else.} \end{cases} \tag{C32}$$

We can solve for the resulting steady-state probability distribution, which is

$$p(\theta) = \frac{M \exp\left(\frac{M(\pi - |\theta|)}{D_r + D_c}\right)}{2(D_r + D_c)\left(\exp\left(\frac{M\pi}{D_r + D_c}\right) - 1\right)}. \tag{C33}$$

The velocity and information rate are similarly analytically tractable, and are given by

$$v/v_0 = \frac{M^2 \coth\left(\frac{M\pi}{2(D_r + D_c)}\right)}{M^2 + (D_r + D_c)^2}, \tag{C34a}$$

$$i = \frac{M^2}{4D_c}. \tag{C34b}$$

Combining the two to eliminate M, we obtain the full pareto frontier

$$v/v_0 = \frac{\coth\left(\frac{\pi}{2}\sqrt{\frac{i}{D_r}}\right)}{1 + \frac{D_r}{i}}. \tag{C35}$$

For low information, the limiting behavior is

$$v/v_0 \sim \frac{2}{\pi}\sqrt{i/D_r}, \tag{C36}$$

while at high information we have

$$v/v_0 \sim 1 - \frac{D_r}{i}. \tag{C37}$$

At low information, this has performance only slightly below that of the full solution (coefficient $2/\pi \approx 0.64$ vs. $1/\sqrt{2} \approx 0.71$), while at high information, it needs twice as much information to obtain the same velocity.

C.6 Steering without sign $\mu(|\theta|)$, at low information rate

We now consider a more constrained case of the continuous steering problem, where the drift $\mu(\theta)$ is required to be an even function of θ. In this case the steady-state distribution no longer takes the Boltzmann form, and there may generally be a net circular flux J. The FPE is (setting $D = D_c + D_r$)

$$J = -\mu(\theta)p(\theta) + Dp'(\theta). \tag{C38}$$

Integrating once more reveals that $2\pi J = -\langle\mu\rangle$. Solving for $\mu(\theta)$ as a function of $p(\theta)$, we have

$$\mu(\theta) = -\frac{J}{p(\theta)} + D\frac{p'(\theta)}{p(\theta)}. \tag{C39}$$

We expand around the low information limit, where μ and $p(\theta)$ are both constant so that up-gradient velocity is zero. For large γ and requiring $\mu(\theta)$ to take the form of a cosine series to enforce evenness, we obtain

$$\mu(\theta) = \pm 2D_r \mp \frac{1}{2\gamma}\cos(\theta). \tag{C40a}$$

The corresponding velocity and information rate are

$$v/v_0 = \frac{1}{16\gamma D_r}, \tag{C41a}$$

$$i = \frac{1}{32\gamma^2 D_r}, \tag{C41b}$$

so that the final scaling is

$$\frac{v}{v_0} = \sqrt{\frac{i}{8D_r}}. \tag{C42}$$

We can also compute the average velocity perpendicular to the gradient, which we find is equal in magnitude to the up-gradient velocity.

Appendix D. Discreteness of the optimal target distribution

For this section, we consider arbitrary jumping strategies. These are specified by the jump rate $\lambda(\theta)$ and the target jump distributions $q_\theta(\Delta\theta)$. While we are allowing the distributions to vary over θ, we focus on the case of symmetric jump distributions, such that $q_\theta(\Delta\theta) = q_\theta(-\Delta\theta)$. Under this specification, the master equation becomes

$$\frac{dp(\theta)}{dt} = -\lambda(\theta)p(\theta) + \int d\Delta\theta\, \lambda(\theta - \Delta\theta)p(\theta - \Delta\theta)q_{\theta-\Delta\theta}(\Delta\theta) + D_r p''(\theta). \tag{D1}$$

Using the above master equation as a constraint, our optimization problem can be written as the following augmented problem:

$$\max_{p,\lambda,(q_\theta)_\theta} \int d\theta\, p(\theta)\cos(\theta) - \gamma i \quad \text{s.t.} \quad -\lambda(\theta)p(\theta) + \int d\Delta\theta\, \lambda(\theta-\Delta\theta)p(\theta-\Delta\theta)q_{\theta-\Delta\theta}(\Delta\theta) + D_r p''(\theta) = 0 \quad \forall\theta, \tag{D2}$$

where p and q_θ are subject to normalization constraints, the distributions q_θ are symmetric, and all choice variables are non-negative.

To invoke some useful results from measure theory, it is convenient to write the target distributions in terms of their corresponding measures. Let Q_θ be the measure associated to the target distribution at θ, such that

$$dQ_\theta(\Delta\theta) = q_\theta(\Delta\theta)\, d\Delta\theta. \tag{D3}$$

Now, let $\langle\lambda\rangle := \int d\theta\, p(\theta)\lambda(\theta)$. Note that $p(\theta)\lambda(\theta)/\langle\lambda\rangle$ is the density of the distribution of locations where jumps are originated. Therefore, the probability of jump sizes averaged over jump sources is captured by the mixture $\bar{Q}$:

$$\bar{Q} := \frac{1}{\langle\lambda\rangle}\int d\theta\, p(\theta)\lambda(\theta)Q_\theta. \tag{D4}$$

Let us denote the collection of target measures with $\mathbf{Q} := (Q_\theta)_\theta$. We can write our information rate in terms of $(p, \lambda, \mathbf{Q})$ as

$$i(p,\lambda,\mathbf{Q}) = \int d\theta\, p(\theta)\lambda(\theta)\left[\log\left(\frac{\lambda(\theta)}{\langle\lambda\rangle}\right) + D_{\mathrm{KL}}(Q_\theta\|\bar{Q})\right] \tag{D5}$$

In terms of the target measures, the Lagrangian of the problem (D2) is

$$\begin{aligned}\mathcal{L}_1(p,\lambda,\mathbf{Q}) = &\int d\theta\, p(\theta)[\cos(\theta) + D_r\chi''(\theta) + \psi_p] \\ &+ \int d\theta\, p(\theta)\lambda(\theta)\left[-\gamma\log\left(\frac{\lambda(\theta)}{\langle\lambda\rangle}\right) - \gamma D_{\mathrm{KL}}(Q_\theta\|\bar{Q}) + \int dQ_\theta(\Delta\theta)\,\chi(\theta+\Delta\theta) - \chi(\theta)\right],\end{aligned} \tag{D6}$$

where ψ_p is the Lagrange multiplier that enforces the normalization of p and $\chi(\theta)$ enforces the master equation constraint. We now separate the problem of optimization over target measures $\mathbf{Q}$ and optimization over (p, λ).

D.1 Optimization over target measures

For fixed p and λ, we can focus on the elements of the Lagrangian $\mathcal{L}_1$ that depend on the measures $\mathbf{Q}$. This inner problem is similar to the one studied in [7] in the context of rational inattention.

Within $\mathcal{L}_1$, the only parts that depend on $\mathbf{Q}$ can be grouped into the following "inner" Lagrangian:

$$\mathcal{L}_2(\mathbf{Q}) := \int d\theta\, p(\theta)\lambda(\theta)\left[\int dQ_\theta(\Delta\theta)\,\chi(\theta+\Delta\theta) - \gamma D_{\mathrm{KL}}(Q_\theta\|\bar{Q})\right]. \tag{D7}$$

Therefore, for a fixed p and λ, optimizing $\mathcal{L}_1$ over $\mathbf{Q}$ is equivalent to solving

$$\max_{\mathbf{Q}} \mathcal{L}_2(\mathbf{Q}) \quad \text{subject to } Q_\theta \text{ symmetric } \forall\theta. \tag{D8}$$

As we show in Section D.6, this problem is equivalent to solving the following augmented problem (for which a symmetric solution always exists):

$$\max_{\mathbf{Q},R} \mathcal{L}_2'(\mathbf{Q}, R), \tag{D9}$$

where now $(Q_\theta)_\theta$ and R are unrestricted probability measures, and we have defined the augmented Lagrangian

$$\mathcal{L}_2'(\mathbf{Q}, R) := \int d\theta\, p(\theta)\lambda(\theta) \left[\frac{1}{2}\int dQ_\theta(\Delta\theta)\left[\chi(\theta+\Delta\theta)+\chi(\theta-\Delta\theta)\right] - \gamma D_{\mathrm{KL}}(Q_\theta\|R)\right]. \tag{D10}$$

As we will show, at the optimum this problem satisfies $R^* = \bar{Q}^*$, which is necessary for these problems to be equivalent.

Let us first do the optimization in (D9) over the measures $\mathbf{Q}$. Note that this can be done independently for each θ by solving the problem

$$\max_{Q_\theta} \mathcal{L}_\theta(Q_\theta), \quad \mathcal{L}_\theta(Q_\theta) := \frac{1}{2}\int dQ_\theta(\Delta\theta)\left[\chi(\theta+\Delta\theta)+\chi(\theta-\Delta\theta)\right] - \gamma D_{\mathrm{KL}}(Q_\theta\|R). \tag{D11}$$

Using the Donsker-Varadhan variational formula for the KL divergence, this problem can be solved in closed form. The unique maximizer (given R) $Q^*_{\theta,R}$ satisfies

$$dQ^*_{\theta,R}(\Delta\theta) = \frac{e^{[\chi(\theta+\Delta\theta)+\chi(\theta-\Delta\theta)]/2\gamma}}{Z_R(\theta)}\, dR(\Delta\theta). \tag{D12}$$

Furthermore, the value of our objective function is

$$\mathcal{L}_\theta(Q^*_{\theta,R}) = \gamma \log\Bigg(\underbrace{\int dR(\Delta\theta)\, e^{\frac{1}{2\gamma}[\chi(\theta+\Delta\theta)+\chi(\theta-\Delta\theta)]}}_{=:Z_R(\theta)}\Bigg). \tag{D13}$$

Using the solution to our optimization of $\mathcal{L}_\theta$, we can return to the problem (D9) and maximize over R. Note that

$$\mathcal{L}_2'(\mathbf{Q}^*_R, R) = \int d\theta\, p(\theta)\lambda(\theta)\mathcal{L}_\theta(Q^*_{\theta,R}) = \gamma \int d\theta\, p(\theta)\lambda(\theta)\log(Z_R(\theta)). \tag{D14}$$

Therefore, our optimization over R is

$$\max_R \mathcal{L}_2'(\mathbf{Q}_R, R) = \max_R \left\{\gamma \int d\theta\, p(\theta)\lambda(\theta)\log(Z_R(\theta))\right\}. \tag{D15}$$

In order to solve problem (D15), we use the technical result in Section D.7 on optimization over measures. First, note that the functional $\mathcal{L}_2'(\mathbf{Q}_R, R)$ is concave, since R enters linearly into the logarithm. We also have the following functional derivative (letting $r(\Delta\theta)$ be the density of R):

$$\frac{\delta \mathcal{L}_2'(\mathbf{Q}_R, R)}{\delta r(\Delta\theta)} = \gamma \int d\theta\, p(\theta)\lambda(\theta)\frac{e^{[\chi(\theta+\Delta\theta)+\chi(\theta-\Delta\theta)]/2\gamma}}{Z_R(\theta)} =: \gamma\langle\lambda\rangle\Psi_R(\Delta\theta). \tag{D16}$$

The function Ψ_R is called the *contact function*, and it determines the support of the optimizer. Using the result in Section D.7, we have that there exists a constant κ such that, at the optimizer R^*,

$$\gamma\langle\lambda\rangle\Psi_{R^*}(\Delta\theta) \le \kappa \quad \forall\Delta\theta, \quad \gamma\langle\lambda\rangle\Psi_{R^*}(\Delta\theta) = \kappa \quad \forall\Delta\theta \in \mathrm{supp}(R^*). \tag{D17}$$

Integrating the second equality against R^* and using Eq. (D12) implies that $\kappa = \gamma\langle\lambda\rangle$. Therefore, our contact conditions are

$$\Psi_{R^*}(\Delta\theta) \le 1 \quad \forall\Delta\theta, \quad \Psi_{R^*}(\Delta\theta) = 1 \quad \forall\Delta\theta \in \mathrm{supp}(R^*). \tag{D18}$$

We can now confirm that this solution satisfies $R^* = \bar{Q}^*$. Using the solution in Eq. (D12), we see that the density of $\bar{Q}^*$ is

$$\begin{aligned} d\bar{Q}^*(\Delta\theta) &= \frac{1}{\langle\lambda\rangle}\int d\theta\, p(\theta)\lambda(\theta) dQ^*_\theta(\Delta\theta) \\ &= \frac{1}{\langle\lambda\rangle}\int d\theta\, p(\theta)\lambda(\theta)\frac{e^{[\chi(\theta+\Delta\theta)+\chi(\theta-\Delta\theta)]/2\gamma}}{Z_{R^*}(\theta)}\, dR^*(\Delta\theta) \\ &= \Psi_{R^*}(\Delta\theta)\, dR^*(\Delta\theta). \end{aligned} \tag{D19}$$

Using our contact conditions, we know that $\Psi_{R^*} = 1$ for all $\Delta\theta$ in the support of R^*. Thus, we have $\bar{Q}^* = R^*$.

Having characterized the optimal target measures, we can return to the optimization over p and λ.

D.2 Optimization over p and λ

Now we consider fixed χ and $\mathbf{Q}$, and optimize the original Lagrangian (D6) over p and λ. Optimizing over λ yields

$$\frac{\delta \mathcal{L}_1(p,\lambda,\mathbf{Q})}{\delta \lambda(\theta)} = p(\theta)\left\{-\gamma \log\left(\frac{\lambda(\theta)}{\langle\lambda\rangle}\right) - \gamma D_{\mathrm{KL}}(Q_\theta \| \bar{Q}) + \int dQ_\theta(\Delta\theta)\,\chi(\theta+\Delta\theta) - \chi(\theta)\right\} = 0. \tag{D20}$$

Similarly, optimizing over p gives

$$\begin{aligned}\frac{\delta \mathcal{L}_1(p,\lambda,\mathbf{Q})}{\delta p(\theta)} &= \cos(\theta) - \gamma\lambda(\theta)\left[\log\left(\frac{\lambda(\theta)}{\langle\lambda\rangle}\right) + D_{\mathrm{KL}}(Q_\theta\|\bar{Q}) - 1\right] + \psi_p + D_r \chi''(\theta) \\ &\quad + \lambda(\theta)\left[\int dQ_\theta(\Delta\theta)\,\chi(\theta+\Delta\theta) - \chi(\theta)\right] = 0.\end{aligned} \tag{D21}$$

Combining these two first-order conditions yields the following second-order differential equation for χ:

$$D_r\chi''(\theta) + \psi_p + \cos(\theta) + \gamma\lambda(\theta) = 0. \tag{D22}$$

Integrating over $[-\pi,\pi]$ and using periodicity yields

$$\psi_p = -\gamma \underbrace{\frac{1}{2\pi}\int d\theta\,\lambda(\theta)}_{=:\lambda_0}. \tag{D23}$$

Therefore, the differential equation for χ is fully characterized by λ:

$$D_r\chi''(\theta) + \cos(\theta) + \gamma[\lambda(\theta) - \lambda_0] = 0. \tag{D24}$$

Additionally, note that for symmetric measures $\mathbf{Q}$, the first-order condition for λ can be written as

$$-\gamma\log\left(\frac{\lambda(\theta)}{\langle\lambda\rangle}\right) - \gamma D_{\mathrm{KL}}(Q_\theta\|\bar{Q}) + \frac{1}{2}\int dQ_\theta(\Delta\theta)\,[\chi(\theta+\Delta\theta)+\chi(\theta-\Delta\theta)] - \chi(\theta) = 0 \tag{D25}$$

Together, Equations (D24) and (D25) capture the structure of the optimal p and λ. We can now put together the $\mathbf{Q}$ and (p,λ) optimizations.

D.3 Joint optimization

Under the optimal measures Q^*_θ, following Eq. (D12), the Donsker-Varadhan result gave us

$$\frac{1}{2}\int dQ^*_\theta(\Delta\theta)\,[\chi(\theta+\Delta\theta)+\chi(\theta-\Delta\theta)] - \gamma D_{\mathrm{KL}}(Q^*_\theta\|\bar{Q}^*) = \gamma\log\left(Z_{\bar{Q}^*}(\theta)\right). \tag{D26}$$

Comparing with Equation (D25), we get

$$Z_{\bar{Q}^*}(\theta) = \frac{\lambda(\theta)}{\langle\lambda\rangle}e^{\chi(\theta)/\gamma}. \tag{D27}$$

This implies that our contact function at the optimum is

$$\Psi_{\bar{Q}^*}(\Delta\theta) = \int d\theta\, p(\theta) e^{[\chi(\theta+\Delta\theta)+\chi(\theta-\Delta\theta)-2\chi(\theta)]/2\gamma}. \tag{D28}$$

We are now ready to prove that the optimal target measures are discrete.

D.4 Discreteness of target support

To show discreteness of the distribution of targets, we show that the contact function can only equal 1 at a finite number of points. We do this by showing that it is analytic and non-constant, as has been done to show discreteness of optimal priors [8].

Now we evaluate all quantities at the optimum. To simplify notation, we define $\Psi := \Psi_{\bar{Q}^*}$. First, note that χ solves the ODE in equation (D24). For solutions with analytic λ (which we implicitly constrain our optimization to), this implies that χ is analytic. Since e^x is an analytic function, it follows that Ψ is analytic in $\Delta\theta$.

Now, note that

$$\Psi''(0) = \frac{1}{\gamma}\int d\theta\, p(\theta)\chi''(\theta). \tag{D29}$$

Taking the master equation (D1), multiplying by $\chi(\theta)$ and integrating yields

$$D_r \int d\theta\, p(\theta)\chi''(\theta) + \int d\theta\, p(\theta)\lambda(\theta)\left[\int dQ_\theta(\Delta\theta)\,\chi(\theta+\Delta\theta) - \chi(\theta)\right] = 0. \tag{D30}$$

Using the first-order condition for λ (D20), we can rewrite the second integral as

$$D_r \int d\theta\, p(\theta)\chi''(\theta) + \gamma\int d\theta\, p(\theta)\lambda(\theta)\left[\log\left(\frac{\lambda(\theta)}{\langle\lambda\rangle}\right) + D(Q_\theta\|\bar{Q})\right] = 0. \tag{D31}$$

Note that this second term is simply the information rate. Therefore,

$$\Psi''(0) = \frac{1}{\gamma}\int d\theta\, p(\theta)\chi''(\theta) = -\frac{i}{D_r} < 0, \tag{D32}$$

since for finite γ the solution achieves a strictly positive information rate. Therefore, Ψ is not constant. Along with analyticity, this means that the contact condition $\Psi(\Delta\theta) = 1$ can only hold at a finite number of points. Thus, the support of the distribution of targets at the optimum must be discrete.

D.5 Contact function for steering strategies

As with jumping strategies, the optimal steering problem can also be formulated in terms of an optimization problem over p, μ and D_c, where the master equation is enforced through the Lagrange multiplier $\chi(\theta)$. The Lagrangian for the steering problem is

$$\mathcal{L}_{\text{steering}}(p,\mu,D_c) = \int d\theta\, p(\theta)\left[\cos(\theta) - \frac{\gamma}{4D_c}[\mu(\theta)-\langle\mu\rangle]^2 + \psi_p\right] + \int d\theta\,\chi(\theta)\left[(D_r+D_c)p''(\theta) - \frac{d}{d\theta}(\mu(\theta)p(\theta))\right], \tag{D33}$$

where ψ_p enforces the normalization constraint for p. For a fixed χ, the optimization problem can be re-written as

$$\max_{p,\mu,D_c}\int d\theta\, p(\theta)\left\{\cos(\theta) - \frac{\gamma}{4D_c}[\mu(\theta)-\langle\mu\rangle]^2 + \psi_p + (D_r+D_c)\chi''(\theta) + \mu(\theta)\chi'(\theta)\right\}. \tag{D34}$$

The first-order condition for μ is

$$\frac{\delta\mathcal{L}_{\text{steering}}(p,\mu,D_c)}{\delta\mu(\theta)} = p(\theta)\chi'(\theta) - \frac{\gamma}{2D_c}p(\theta)[\mu(\theta)-\langle\mu\rangle] = 0. \tag{D35}$$

Here we can invoke the solution to the problem from Section C.2. We have $D_c^* = D_r$, $\langle\mu\rangle = 0$ and $\mu(\theta) = 2D_r\partial_\theta\log(p(\theta))$. Using this in our differential equation for χ yields

$$\chi'(\theta) = \gamma\partial_\theta\log(p(\theta)) \quad\Longrightarrow\quad \frac{\chi(\theta)}{\gamma} = \log(p(\theta)) + c_0, \tag{D36}$$

for some constant c_0.

Since the contact function for jump strategies in Section D.3 only depends on the Lagrange multiplier χ, we can evaluate it for the steering case. For jumping strategies with non-symmetric targets Q_θ, the contact function would be

$$\Psi(\Delta\theta) = \int d\theta\, p(\theta) e^{[\chi(\theta+\Delta\theta)-\chi(\theta)]/\gamma}. \tag{D37}$$

With our solution for χ in the steering case, we can evaluate the contact function to be

$$\Psi(\Delta\theta) = \int d\theta\, p(\theta+\Delta\theta) = 1. \tag{D38}$$

D.6 Equivalence of the augmented optimization problem

Let us consider the problem

$$\begin{aligned} &\max_{\mathbf{Q}} \mathcal{L}_2(\mathbf{Q}) \quad \text{subject to } Q_\theta \text{ symmetric } \forall\theta, \\ &\mathcal{L}_2(\mathbf{Q}) := \int d\theta\, p(\theta)\lambda(\theta) \left[\int dQ_\theta(\Delta\theta)\, \chi(\theta+\Delta\theta) - \gamma D_{\mathrm{KL}}(Q_\theta \| \bar{Q})\right], \end{aligned} \tag{D39}$$

Since we are considering symmetric measures, solving the problem above is the same as solving it for the symmetrized objective

$$\mathcal{L}_2^s(\mathbf{Q}) = \int d\theta\, p(\theta)\lambda(\theta) \left[\frac{1}{2}\int dQ_\theta(\Delta\theta)\, [\chi(\theta+\Delta\theta) + \chi(\theta-\Delta\theta)] - \gamma D_{\mathrm{KL}}(Q_\theta \| \bar{Q})\right]. \tag{D40}$$

We will now prove that the optimization problem with the objective function (D40) can be solved with symmetric measures, even when the symmetry constraint is lifted. When the symmetry constraint is lifted, the space of admissible measures is expanded, so if the solution of the expanded problem is symmetric, it will also be a solution of the constrained problem. That is, we now consider the problem

$$\max_{\mathbf{Q}} \mathcal{L}_2^s(\mathbf{Q}). \tag{D41}$$

Consider an arbitrary collection $\mathbf{Q}$ (not necessarily symmetric). We will prove that symmetrizing it weakly improves the objective function $\mathcal{L}_2^s$. Define the symmetrized measures

$$Q_\theta^s := \frac{1}{2}(Q_\theta + Q_\theta^-), \tag{D42}$$

where Q_θ^- is the measure obtained under the transformation $\Delta\theta \to -\Delta\theta$. Clearly these measures satisfy

$$\int dQ_\theta(\Delta\theta)\, [\chi(\theta+\Delta\theta) + \chi(\theta-\Delta\theta)] = \int dQ_\theta^s(\Delta\theta)\, [\chi(\theta+\Delta\theta) + \chi(\theta-\Delta\theta)]. \tag{D43}$$

Therefore, the first part of the objective is unchanged under symmetrization. Additionally, using the convexity of the KL divergence, we have that

$$D_{\mathrm{KL}}(Q_\theta^s \| \bar{Q}^s) \leq D_{\mathrm{KL}}(Q_\theta \| \bar{Q}) \quad \forall\theta. \tag{D44}$$

This means that symmetrizing the measures weakly improves the objective:

$$\mathcal{L}_2^s(\mathbf{Q}^s) \geq \mathcal{L}_2^s(\mathbf{Q}) \quad \forall\mathbf{Q}. \tag{D45}$$

Therefore, if a non-symmetric solution exists, it can always be symmetrized to weakly improve the objective. We can, then, find our optimal measures by solving the unconstrained problem.

To make progress, we again augment the problem by optimizing over an additional probability measure R:

$$\max_{\mathbf{Q},R} \mathcal{L}_2'(\mathbf{Q}, R), \tag{D46}$$

where the augmented Lagrangian is

$$\mathcal{L}_2'(\mathbf{Q}, R) := \int d\theta\, p(\theta)\lambda(\theta) \left[\frac{1}{2}\int dQ_\theta(\Delta\theta)\,[\chi(\theta+\Delta\theta)+\chi(\theta-\Delta\theta)] - \gamma D_{\mathrm{KL}}(Q_\theta \| R)\right]. \tag{D47}$$

Note that this augmented Lagrangian satisfies

$$\mathcal{L}_2'(\mathbf{Q}, \bar{Q}) = \mathcal{L}_2^s(\mathbf{Q}). \tag{D48}$$

This means that our search space reaches all possible values of the optimization problem (D41). Now, suppose a solution to (D46) problem exists that satisfies

$$R^* = \frac{1}{\langle\lambda\rangle}\int d\theta\, p(\theta)\lambda(\theta) Q_\theta^* = \bar{Q}^*. \tag{D49}$$

This would imply that $\mathbf{Q}^*$ also solves (D41). This condition is verified in (D19), so our augmented problem (D46) also solves our original problem (D39).

D.7 Optimization over measures

In this section, we prove a technical result about the support of the solution when optimizing over measures.

Lemma.— Let $\mathcal{X}$ be a compact metric space and $\mathcal{P}(\mathcal{X})$ be the set of probability measures over $\mathcal{X}$. Suppose the function $F : \mathcal{P}(\mathcal{X}) \to \mathbb{R}$ is concave and that there exists $f : \mathcal{P}(\mathcal{X}) \times \mathcal{X} \to \mathbb{R}$ such that

$$\frac{d}{dt}F((1-t)\mu + t\nu)\Big|_{t=0^+} = \int_{\mathcal{X}} f_\mu(x)\, d(\nu-\mu)(x). \tag{D50}$$

Additionally, suppose f is continuous on x for all μ. Then for any μ^* that solves the problem

$$\max_{\mu\in\mathcal{P}(\mathcal{X})} F(\mu) \tag{D51}$$

there exists a constant κ such that

$$f_{\mu^*}(x) \le \kappa \quad \forall x \in \mathcal{X}, \qquad f_{\mu^*}(x) = \kappa \quad \forall x \in \mathrm{supp}(\mu^*). \tag{D52}$$

Proof. Fix $\nu \in \mathcal{P}(\mathcal{X})$ and a maximizer μ^*. Define the function $\phi : [0,1] \to \mathbb{R}$ as

$$\phi(t) := F((1-t)\mu^* + t\nu). \tag{D53}$$

By concavity of F, the function ϕ is concave. Additionally, since μ^* is a maximizer of F, we have that ϕ must attain its maximum at $t = 0$. For a concave function that attains its maximum at its left endpoint, we must have $\phi'(0^+) \le 0$. By Eq. (D50), this implies that

$$\int_{\mathcal{X}} f_{\mu^*}(x)\, d\nu(x) \le \int_{\mathcal{X}} f_{\mu^*}(x)\, d\mu^*(x) =: \kappa. \tag{D54}$$

Note that this bound holds for arbitrary measures. In particular, taking the Dirac measure at x_0,

$$\int_{\mathcal{X}} f_{\mu^*}(x)\, d\delta_{x_0}(x) = f_{\mu^*}(x_0) \le \kappa. \tag{D55}$$

Additionally, note that the maximum over ν of $\int_{\mathcal{X}} f_{\mu^*}(x)\, d\nu(x)$ is attained when the measure ν concentrates its mass on the maxima of f_{μ^*}. Since the inequality in Eq. (D54) holds for all ν, it must be the case that μ^* attains this maximum, so it must also concentrate its mass on the maxima of f_{μ^*}. This implies that

$$\kappa = \int_{\mathcal{X}} f_{\mu^*}(x)\, d\mu^*(x) = \max_{x\in\mathcal{X}} f_{\mu^*}(x). \tag{D56}$$

Therefore, we must have $f_{\mu^*}(x) = \kappa$ μ^*-almost everywhere. Since f_{μ^*} is continuous, this implies the stronger result that $f_{\mu^*}(x) = \kappa$ for all $x \in \mathrm{supp}(\mu^*)$. □

The result above generalizes the usual KKT conditions for constrained optimization to the case of measures. Here, the inequality $f_{\mu^*}(x) \le \kappa$ ensures the non-negativity constraint of the measure. For points on the support, the constraint is slack, and the constraint binds for all points where the inequality is strict.

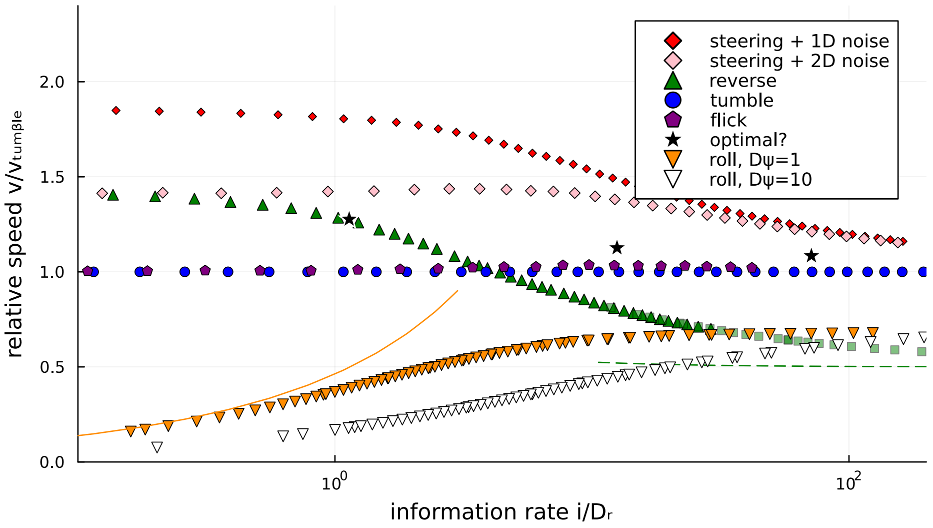

Figure S5: Performance of strategies for three-dimensional navigation, relative to tumbling. Red and pink diamonds show continuous steering with directional information for both "1D noise" (red) and "2D noise" (pink). Green triangles, blue circles, and purple pentagons respectively show the reverse, tumble, and flick strategies, while black stars indicate the optimal discrete strategy with only scalar information. Inverted triangles show scalar steering with roll diffusion, with $D_\psi = D_r$ (orange) and $D_\psi = 10D_r$ (white). Green dashed line shows the asymptotic scaling of the tumble strategy in the high information limit, while the orange solid line shows the conjectured scaling of the unsigned?? steering strategy in the low information limit.

Appendix E. Navigation in three or more dimensions

In the main text we briefly discussed how the navigation problem and resulting optimal strategies change in three dimensions. Here we provide the details.

E.1 Simple strategies in $d = 3$

Allowing steering and tumbles, the Fokker-Planck equation on the sphere of possible headings reads

$$\frac{dp}{dt} = -\vec{\nabla} \cdot (\vec{\mu} p) - \lambda p + \langle \lambda \rangle + D\nabla^2 p. \tag{E1}$$

Assuming radial symmetry $p(\theta, \phi) = p(\theta)$, and allowing complete tumbles, where the new heading is uniform on the sphere, we have:

$$\frac{dp(\theta)}{dt} = \underbrace{\int d\theta' \sin\theta' p(\theta')\lambda(\theta') - \lambda(\theta)p(\theta)}_{\text{tumbles}} + \underbrace{D_r \frac{1}{\sin\theta}\frac{d}{d\theta}\left[\sin\theta \frac{dp(\theta)}{d\theta}\right]}_{\text{diffusion}}. \tag{E2}$$

The simplest kind of steering is only towards or away from the North pole ($\theta = 0$). The appropriate controller noise D_c is one-dimensional diffusion along the line of latitude, $\frac{d}{dt}(\sin\theta\, p(\theta)) = D_c \frac{d^2}{d\theta^2}(\sin\theta\, p(\theta))$. This leads to the following Fokker-Planck equation:

$$\frac{dp(\theta)}{dt} = -\frac{1}{\sin\theta}\frac{d}{d\theta}\left[\sin\theta \mu(\theta)p(\theta)\right] + \frac{1}{\sin\theta}\frac{d}{d\theta}\left[(D_r + D_c)\sin\theta \frac{dp(\theta)}{d\theta} + D_c \cos\theta\, p(\theta)\right]. \tag{E3}$$

This is described as "1D noise" on the figures, and gives the red points in figure 4 in the main text. But another, simpler, choice of controller noise is to add another term exactly like the rotational diffusion term D_r:

$$\frac{dp(\theta)}{dt} = -\frac{1}{\sin\theta}\frac{d}{d\theta}\left[\sin\theta \mu(\theta)p(\theta)\right] + \frac{1}{\sin\theta}\frac{d}{d\theta}\left[(D_r + D_c)\sin\theta \frac{dp(\theta)}{d\theta}\right]. \tag{E4}$$

This choice is described as "2D noise" on the figures, and the resulting pink points have slightly lower performance.

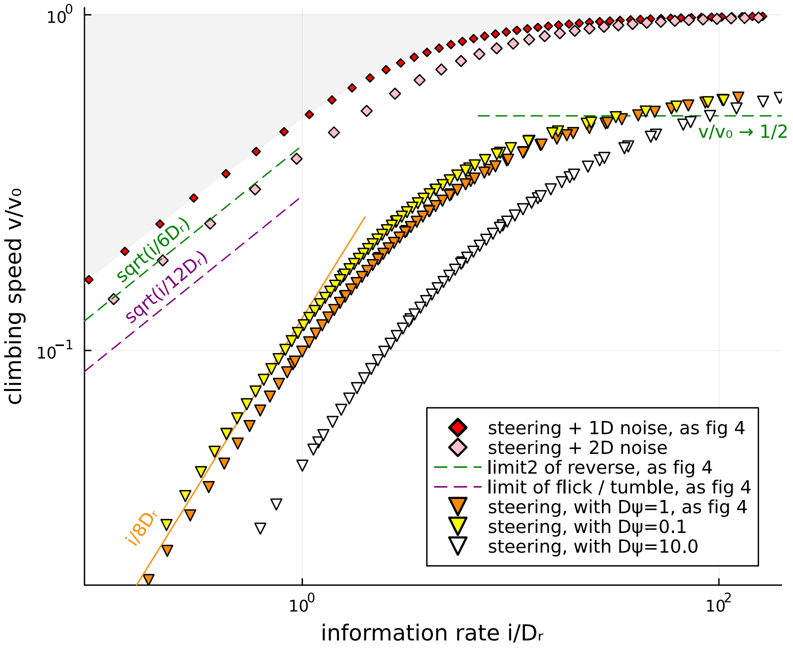

Figure S6: More results for 3D steering strategies, with velocity normalized by the maximum swimming speed. Red and pink diamonds show continuous steering with directional information for both "1D noise" (red) and "2D noise" (pink). Inverted triangles show scalar steering with roll diffusion, with $D_\psi = D_r$ (orange), $D_\psi = 0.1 D_r$ (yellow), and $D_\psi = 10 D_r$ (white). Green and purple dashed lines show the low- and high-information scaling for tumble and flick respectively, while the orange solid line shows the conjectured low-information scaling for scalar steering.

We could also allow for both reversing the heading at rate $\zeta(\theta)$ and 90 degree flicks at rate $\kappa(\theta)$. The sink terms are identical, but the source term involves an integral around a circle perpendicular to the original heading vector. Integrating out the azimuthal angle ϕ, we obtain:

$$\frac{dp(\theta)}{dt} = \text{previous} - \underbrace{\zeta(\theta)p(\theta) + \zeta(\pi-\theta)p(\pi-\theta)}_{\text{reverse with rate } \zeta} - \underbrace{\kappa(\theta)p(\theta) + \frac{1}{2\pi}\int_0^{2\pi} d\psi\, \kappa(\theta'(\psi,\theta))p(\theta'(\psi,\theta))}_{\text{flick with rate } \kappa} \tag{E5}$$

where $\theta'(\psi,\theta) = \arccos(-\cos\psi \sin\theta)$.

Figures S5 and S6 shows the performance trade-off curves for several of these strategies, computed from numerical solutions to the Fokker-Planck equation. As with the two-dimensional case we find that directed steering is optimal, although now the choice between (E3) and (E4) gives two different results. Among strategies not using the direction, The reverse strategy performs well in the low information limit, scaling as $v/v_0 \approx \sqrt{i/6D_r}$. Both tumble and flick perform worse, scaling in the low information limit as $v/v_0 \approx \sqrt{i/12D_r}$, but both outperform reverse at high information. The reverse strategy reaches a maximum velocity of $v/v_0 = 1/2$ at high information, while the flick and tumble strategies reach $v/v_0 = 1$ in the same limit.

E.2 Information rate for steering in d dimensions

While the generalization of the information rate is straightforward for discrete jumps, the generalization for continuous steering is somewhat more involved. Here we derive the information rate for the full d-dimensional version of the navigation problem. With only continuous steering, the dynamics for the heading vector $\boldsymbol{n}$ on the $d-1$ dimensional sphere are

$$\dot{\boldsymbol{n}} = \boldsymbol{\mu}(\boldsymbol{n}) + \sqrt{2(D_r + D_c)}\boldsymbol{\eta}(t), \tag{E6}$$

with $|\boldsymbol{n}| = 1$ enforced for all t. Note that here we make the choice of "2D noise" as described in the previous section. The steering force $\boldsymbol{\mu}$ resides in the $d-1$ dimensional space tangent to the $d-1$ sphere at the point $\boldsymbol{n}$, as does the random force $\boldsymbol{\eta}(t)$.

We are interested in computing the information rate

$$i = \lim_{\mathrm{d}t \to 0} \frac{I[\boldsymbol{n}; \boldsymbol{\Delta_c n}]}{\mathrm{d}t}, \tag{E7}$$

where

$$\boldsymbol{\Delta_c n} := \boldsymbol{\mu}(\boldsymbol{n})\mathrm{d}t + \sqrt{2D_c \mathrm{d}t}\,\boldsymbol{\eta}(t), \tag{E8}$$

where $\boldsymbol{\eta}$ has mean 0 and covariance given by the $d-1$ dimensional identity matrix 1_{d-1}. We then rescale this variable to a zero-mean variant,

$$\boldsymbol{z} := \frac{\boldsymbol{\Delta_c n} - \langle \mu \rangle_{p(\boldsymbol{n})} \mathrm{d}t}{\sqrt{2D_c \mathrm{d}t}}, \tag{E9}$$

whose distribution conditional on $\boldsymbol{n}$ satisfies

$$p(\boldsymbol{z}|\boldsymbol{n}) = \mathcal{N}\left[\sqrt{\frac{\mathrm{d}t}{2D_c}}\left(\boldsymbol{\mu}(\boldsymbol{n}) - \langle \mu \rangle_{p(\boldsymbol{n})}\right), 1_{d-1}\right]. \tag{E10}$$

The marginal distribution $p(\boldsymbol{z})$ has zero mean. Note that rescaling and shifting a variable has no effect on mutual information, so we can rewrite the information rate as

$$i = \lim_{\mathrm{d}t \to 0} \frac{I[\boldsymbol{n}; \boldsymbol{z}]}{\mathrm{d}t}. \tag{E11}$$

We then rewrite the mutual information as a KL divergence and expand, obtaining

$$I[\boldsymbol{n}; \boldsymbol{z}] = \langle D_{\mathrm{KL}}[p(\boldsymbol{z}|\boldsymbol{n}) \| p(\boldsymbol{z})] \rangle_{p(\boldsymbol{n})} \tag{E12a}$$

$$= \langle D_{\mathrm{KL}}[p(\boldsymbol{z}|\boldsymbol{n}) \| \tilde{p}(\boldsymbol{z})] \rangle_{p(\boldsymbol{n})} - D_{\mathrm{KL}}[p(\boldsymbol{z}) \| \tilde{p}(\boldsymbol{z})]. \tag{E12b}$$

Here we have defined

$$\tilde{p}(\boldsymbol{z}) := \mathcal{N}(0, 1_{d-1}). \tag{E13}$$

The first term is straightforward to evaluate since it is a KL-divergence between two Gaussians with equal covariances, and yields

$$\langle D_{\mathrm{KL}}[p(\boldsymbol{z}|\boldsymbol{n}) \| \tilde{p}(\boldsymbol{z})] \rangle_{p(\boldsymbol{n})} = \left\langle \frac{1}{2} |\langle \boldsymbol{z} \rangle_{p(\boldsymbol{z}|\boldsymbol{n})}|^2 \right\rangle_{p(\boldsymbol{n})} \tag{E14a}$$

$$= \left\langle \frac{\mathrm{d}t}{4D_c} |\boldsymbol{\mu}(\boldsymbol{n}) - \langle \boldsymbol{\mu} \rangle_{p(\boldsymbol{n})}|^2 \right\rangle_{p(\boldsymbol{n})} \tag{E14b}$$

$$= \frac{\mathrm{d}t}{4D_c} \mathrm{Tr}\left[\mathrm{Cov}(\boldsymbol{\mu})\right], \tag{E14c}$$

where the covariance is in the $d-1$ dimensional tangent space. It then remains to evaluate the second term. Here we define $\boldsymbol{m}(\boldsymbol{n}) = \langle \boldsymbol{z} \rangle_{p(\boldsymbol{z}|\boldsymbol{n})}$, and note that it is proportional to $\sqrt{\mathrm{d}t}$ and thus small, then note that we can write the marginal distribution as

$$p(z) = \langle \tilde{p}\left(\boldsymbol{z} - \boldsymbol{m}(\boldsymbol{n})\right) \rangle_{p(\boldsymbol{n})} \tag{E15a}$$

$$= \left\langle \tilde{p}\left(\boldsymbol{z}\right) \left[1 + \boldsymbol{z} \cdot \boldsymbol{m}(\boldsymbol{n}) + \frac{1}{2}\left([\boldsymbol{z} \cdot \boldsymbol{m}(\boldsymbol{n})]^2 - |\boldsymbol{m}(\boldsymbol{n})|^2\right) + \mathcal{O}(\boldsymbol{m}^3)\right] \right\rangle_{p(\boldsymbol{n})} \tag{E15b}$$

$$= \tilde{p}(\boldsymbol{z}) \left[1 + 0 + \frac{1}{2}\left(\boldsymbol{z}^\top \Sigma_{\boldsymbol{m}} \boldsymbol{z} - \mathrm{Tr}\Sigma_{\boldsymbol{m}}\right) + \mathcal{O}(\mathrm{d}t^{3/2})\right]. \tag{E15c}$$

Here we expanded around $\boldsymbol{m} = 0$, and kept terms up to first order in $\mathrm{d}t$. We can then evaluate the 2nd KL-divergence term by expanding the integrand around $\mathrm{d}t = 0$, which yields

$$D_{\mathrm{KL}}[p(\boldsymbol{z}) \| \tilde{p}(\boldsymbol{z})] = 0 + \frac{1}{2}\left[\underbrace{\langle \boldsymbol{z}^\top \Sigma_{\boldsymbol{m}} \boldsymbol{z} \rangle_{\tilde{p}(\boldsymbol{z})}}_{\mathrm{Tr}\Sigma_{\boldsymbol{m}}} - \mathrm{Tr}\Sigma_{\boldsymbol{m}}\right] + \mathcal{O}(\mathrm{d}t^{3/2}) = 0 + \mathcal{O}(\mathrm{d}t^{3/2}). \tag{E16}$$

Thus after dividing by dt, this contribution to the mutual information is negligible in the small-dt limit. This concludes our proof, and we have

$$i = \frac{1}{4D_c}\mathrm{Tr}\left[\mathrm{Cov}(\boldsymbol{\mu})\right]. \tag{E17}$$

E.3 Continuous Steering in d Dimensions

For continuous steering, we analytically obtain a near-optimal strategy in d-dimensions with “2D noise”. When signed information is available, we expect an equilibrium stationary distribution (from the dynamics given by Eq. (E6)) such that

$$\boldsymbol{\mu}(\boldsymbol{n}) = (D_r + D_c)\nabla \ln p(\boldsymbol{n}). \tag{E18}$$

Analogous to the von-Mises ansatz in two dimensions, we consider a von-Mises-fisher ansatz here,

$$p(\boldsymbol{n}) = \frac{\kappa^{d/2-1}}{(2\pi)^{d/2}1_{d/2-1}(\kappa)}\exp\left(\kappa \boldsymbol{r}^\top \boldsymbol{n}\right), \tag{E19}$$

where κ is a shape parameter and $\boldsymbol{r}$ is the target heading.

The generalization for the velocity is $v/v_0 = \langle \boldsymbol{n}\cdot\boldsymbol{r}\rangle$, which we can evaluate as

$$v/v_0 = \frac{1_{d/2}(\kappa)}{1_{d/2-1}(\kappa)}, \tag{E20}$$

with $I_m(x)$ denoting the m-th order modified Bessel function of the first kind. We can similarly evaluate the information rate, for which we obtain

$$i = \frac{1}{4D_c}\mathrm{Tr}\left[\mathrm{Cov}(\boldsymbol{\mu})\right], \tag{E21}$$

which can be evaluated as

$$i/D_r = (d-1)\kappa\frac{1_{d/2}(\kappa)}{1_{d/2-1}(\kappa)}. \tag{E22a}$$

Here we have taken $D_c = D_r$, which is optimal by the same arguments we used in two dimensions.

Combining these two results and eliminating κ, we obtain an analytic performance trade-off curve in arbitrary dimensions, which will be a lower bound on the optimal Pareto frontier:

$$v/v_0 = \frac{1_{d/2}\left(\frac{i/D_r}{(d-1)v/v_0}\right)}{1_{d/2-1}\left(\frac{i/D_r}{(d-1)v/v_0}\right)}. \tag{E23}$$

Expanding in the low and high information limits this gives

$$v/v_0 \approx \begin{cases} \sqrt{\frac{i/D_r}{d(d-1)}} & i/D_r \ll 1, \\ 1 - \frac{(d-1)^2}{2i/D_r} & i/D_r \gg 1. \end{cases} \tag{E24}$$

E.4 Undirected Steering in 3 Dimensions

We then turn to the question of how the even steering solutions in 2 dimensions should be generalized to 3 dimensions. Consider the degree of deviation of the current heading $\boldsymbol{n}$ from the target heading $\boldsymbol{r}$, which can be quantified by the angle θ between them such that $\boldsymbol{n}\cdot\boldsymbol{r} = \cos\theta$. To parameterize $\boldsymbol{n}$ we require two angles, θ (the angle between the heading and the target), and ϕ the perpendicular angle with respect to which we expect the problem to be symmetric. Now suppose that the strategy $\boldsymbol{\mu}$ can only depend on the value of $\boldsymbol{n}\cdot\boldsymbol{r}$, or equivalently on $\cos\theta$. What is the optimal strategy under this constraint?

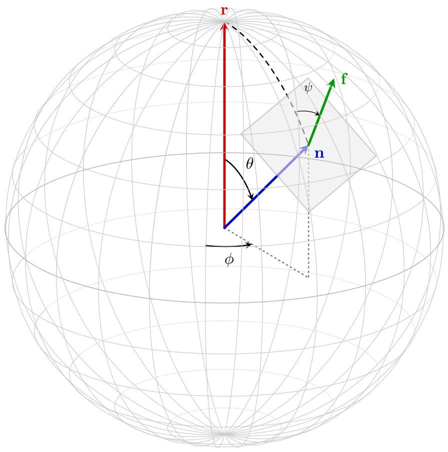

Figure S7: Schematic to illustrate the vectors and angles involved in 3D unsigned steering.

In two dimensions the agent was pinned to a plane, and could thus choose to steer left or right even without knowledge of which direction pointed towards the target direction. In three dimensions, however, this no longer makes sense, since the agent can roll freely around the heading vector $\boldsymbol{n}$. We thus treat the steering capabilities of the agent as applying a steering force in a vector direction $\boldsymbol{f}$ which is perpendicular to $\boldsymbol{n}$, such that $\boldsymbol{\mu} = \boldsymbol{f}\, g(\theta)$ for some scalar function $g(\theta)$ which we require to be even.

To make progress, we define an additional "facing" vector $\boldsymbol{f}$ which is perpendicular to $\boldsymbol{n}$, and is the direction in which the agent can steer. We will quantify $\boldsymbol{f}$ using the angle ψ between $\boldsymbol{f}$ and the optimal steering force direction. We would then write the steering force as $\boldsymbol{\mu} = \boldsymbol{f}\, g(\theta)$. We will describe the dynamics of this steering vector using the angle ψ between $\boldsymbol{f}$ and the tangent vector pointing directly towards the north pole. We assume this angle also undergoes rotational diffusion with a diffusion coefficient D_ψ. Figure S7 illustrates the geometry of the problem.

Carefully keeping track of the relevant geometric and trigonometric factors, we find that three angles in the problem evolve according to the following stochastic differential equations:

$$\dot{\theta} = -g(\theta)\cos\psi + (D_r + D_c)\cot\theta + \sqrt{2(D_c + D_r)}\eta_\theta(t), \tag{E25a}$$

$$\dot{\phi} = -g(\theta)\sin\psi / \sin\theta + \frac{1}{\sin\theta}\sqrt{2(D_c + D_r)}\eta_\phi(t), \tag{E25b}$$

$$\dot{\psi} = g(\theta)\sin\psi\cot\theta + \sqrt{2D_\psi}\eta_\psi(t). \tag{E25c}$$

Here we have applied the controller noise D_c to both the θ and ϕ directions, and treated heading diffusion on the sphere and roll diffusion as independent.

The associated Fokker-Planck equation is

$$\begin{aligned} \frac{\partial}{\partial t} p(\theta,\phi,\psi) = &-\frac{1}{\sin\theta}\partial_\theta\left[-g(\theta)\cos\psi\sin\theta\, p(\theta,\phi,\psi)\right] - \frac{1}{\sin\theta}\partial_\phi\left[-g(\theta)\sin\psi\, p(\theta,\phi,\psi)\right] \\ &- \partial_\psi\left[g(\theta)\cot\theta\sin\psi\, p(\theta,\phi,\psi)\right] + D_\psi\partial_\psi^2 p + (D_r + D_c)\left[\frac{1}{\sin\theta}\partial_\theta\left(\sin\theta\,\partial_\theta p\right) + \frac{1}{\sin^2\theta}\partial_\phi^2 p\right]. \end{aligned} \tag{E26}$$

We then seek to optimize the objective

$$\mathcal{L} = \langle\cos\theta\rangle - \gamma\frac{1}{4D_c}\mathrm{Tr}\left[\mathrm{Cov}(\boldsymbol{\mu})\right] \tag{E27}$$

with respect to D_c and the function $g(x)$.

We will derive the scaling in the low information limit using a series expansion. In the low information limit we have $g_0 = 0$ and $p_0 = 1/8\pi^2$. We now expand around this limit in a small parameter $\epsilon \propto 1/\gamma$, as

$$p = \frac{1}{8\pi^2} + \epsilon p_1 + \mathcal{O}(\epsilon^2), \tag{E28a}$$

$$g = 0 + \epsilon g_1(\theta) + \mathcal{O}(\epsilon^2). \tag{E28b}$$

We require that g_1 be an even function of θ.

We will also assume p is independent of ϕ so that the FPE can be simplified to

$$\frac{\partial}{\partial t}p(\theta,\psi) = -\frac{1}{\sin\theta}\partial_\theta\left[-g(\theta)\cos\psi\sin\theta\, p(\theta,\psi)\right] - \partial_\psi\left[g(\theta)\cot\theta\sin\psi\, p(\theta,\psi)\right] + D_\psi\partial_\psi^2 p + (D_r+D_c)\frac{1}{\sin\theta}\partial_\theta\left(\sin\theta\,\partial_\theta p\right). \tag{E29}$$

At first order, we will have

$$\begin{aligned} 0 =& D_\psi\partial_\psi^2 p_1 + (D_r+D_c)\frac{1}{\sin\theta}\partial_\theta\left(\sin\theta\,\partial_\theta p_1\right) - \frac{1}{\sin\theta}\partial_\theta\left[-g_1\cos\psi\sin\theta\, p_0\right] - \partial_\psi\left[g_1\cot\theta\sin\psi\, p_0\right]. \\ =& D_\psi\partial_\psi^2 p_1 + (D_r+D_c)\left[\partial_\theta^2 p_1 + \cot\theta\,\partial_\theta p_1\right] + \cos\psi\, p_0\,\partial_\theta g_1. \end{aligned} \tag{E30}$$

This functional form strongly suggests the ansatz $p_1(\theta,\psi) = T(\theta)\cos(\psi)$. Any higher harmonics in ψ could exist transiently, but should decay to zero at steady state due to the $\cos\psi$ forcing. This ansatz allows us to write

$$0 = -D_\psi\cos\psi T(\theta) + \cos\psi(D_r+D_c)\left[T''(\theta) + \cot\theta\, T'(\theta)\right] + \frac{1}{8\pi^2}\cos\psi g_1'(\theta). \tag{E31}$$

Dividing through by $\cos\psi$ then gives an ODE for $T(\theta)$,

$$0 = -D_\psi T(\theta) + (D_r+D_c)\left[T''(\theta) + \cot\theta\, T'(\theta)\right] + \frac{1}{8\pi^2}g_1'(\theta). \tag{E32}$$

We can then proceed to evaluate the velocity and information rate. We begin with the information rate, which is

$$\begin{aligned} i &= \frac{1}{4D_c}\mathrm{Tr}\left[\mathrm{Cov}(\boldsymbol{\mu})\right] \\ &= \frac{1}{4D_c}\langle g^2\rangle_{p(\theta,\phi,\psi)} \\ &= \frac{1}{4D_c}\int\int\int \epsilon^2 g_1(\theta)^2 p_0(\theta,\phi,\psi)\sin\theta \mathrm{d}\theta\mathrm{d}\phi\mathrm{d}\psi + \mathcal{O}(\epsilon^3) \\ &= \frac{\epsilon^2}{8D_c}\int g_1(\theta)^2\sin\theta\mathrm{d}\theta + \mathcal{O}(\epsilon^3). \end{aligned} \tag{E33}$$

Since $\sin\theta > 0$ on $(0,\pi)$, so long as g_1 is not identically zero for all θ the information rate will be second order in ϵ. We similarly evaluate the velocity as

$$\begin{aligned} v/v_0 &= \langle\cos\theta\rangle \\ &= \epsilon\int\int\int \cos\theta p_1(\theta,\phi,\psi)\sin\theta\mathrm{d}\theta\mathrm{d}\phi\mathrm{d}\psi + \mathcal{O}(\epsilon^2) \\ &= 2\pi\epsilon\left(\int\cos\psi\mathrm{d}\psi\right)\left(\int T(\theta)\cos\theta\sin\theta\mathrm{d}\theta\right) + \mathcal{O}(\epsilon^2) \\ &= 0 + \mathcal{O}(\epsilon^2). \end{aligned} \tag{E34}$$

Unlike in two dimensions, the up-gradient velocity is exactly zero to first order in ϵ, while the information rate is non-zero at second order in ϵ. Thus $v \sim \sqrt{i}$ scaling is impossible, and the low information velocity-information scaling can be at best $v \sim i$.
